# PI3K-C2α knockdown decreases autophagy and maturation of endocytic vesicles

**DOI:** 10.1371/journal.pone.0184909

**Published:** 2017-09-14

**Authors:** Nathan M. Merrill, Joshua L. Schipper, Jonathan B. Karnes, Audra L. Kauffman, Katie R. Martin, Jeffrey P. MacKeigan

**Affiliations:** 1 Van Andel Institute Graduate School, Grand Rapids, Michigan, United States of America; 2 Center for Cancer Cell Biology, Van Andel Research Institute, Grand Rapids, Michigan, United States of America; 3 College of Human Medicine, Michigan State University, Grand Rapids, Michigan, United States of America; National Heart Lung and Blood Institute, UNITED STATES

## Abstract

Phosphoinositide 3-kinase (PI3K) family members are involved in diverse cellular fates including cell growth, proliferation, and survival. While many molecular details are known about the Class I and III PI3Ks, less is known about the Class II PI3Ks. To explore the function of all eight PI3K isoforms in autophagy, we knock down each gene individually and measure autophagy. We find a significant decrease in autophagy following siRNA-mediated *PIK3C2A* (encoding the Class 2 PI3K, PI3K-C2α) knockdown. This defective autophagy is rescued by exogenous PI3K-C2α, but not kinase-dead PI3K-C2α. Using confocal microscopy, we probe for markers of endocytosis and autophagy, revealing that PI3K-C2α colocalizes with markers of endocytosis. Though endocytic uptake is intact, as demonstrated by transferrin labeling, *PIK3C2A* knockdown results in vesicle accumulation at the recycling endosome. We isolate distinct membrane sources and observe that PI3K-C2α interacts with markers of endocytosis and autophagy, notably ATG9. Knockdown of either *PIK3C2A* or *ATG9A/B*, but not *PI3KC3*, results in an accumulation of transferrin-positive clathrin coated vesicles and RAB11-positive vesicles at the recycling endosome. Taken together, these results support a role for PI3K-C2α in the proper maturation of endosomes, and suggest that PI3K-C2α may be a critical node connecting the endocytic and autophagic pathways.

## Introduction

Macroautophagy (autophagy) is an intracellular degradation pathway that targets cytosolic material for lysosomal degradation [1–3]. Under conditions of stress, such as nutrient starvation, this process is used to produce amino acids and other biochemical building blocks to promote cell survival [4–7]. The importance of autophagy is underscored by its deregulation in a number of diseases, notably cancer [4,8,9]. While considerable progress has been made characterizing the mechanism of this process, many important issues, such as those concerning the incorporation of different membrane sources into this pathway, remain unaddressed.

In response to nutrient stress, key cellular signals, such as the activation of AMP activated protein kinase (AMPK) and suppression of mechanistic target of rapamycin complex 1 (mTORC1), result in the activation of the Unc-51 Like autophagy activating kinase 1/2 (ULK1/2) regulatory complex [10–14]. ULK1/2 is responsible for the initiation of autophagy through the phosphorylation of several necessary autophagic components, including Autophagy Related Protein 9 (ATG9) and the BECLIN1-PI3KC3 complex [15–17]. Phosphorylation of ATG9 promotes binding to the AP1/2 clathrin adaptor complex and, along with BECLIN1-PI3KC3 complex activation, is required for membrane nucleation and initial formation of pre-autophagosomal vesicles [16–20]. Elongation of the phagophore, the initial cup-shaped autophagy membrane, and the recruitment of organelles and proteins targeted for degradation follows, and this is associated with both conjugation of ATG12 to ATG5 and microtubule-associated protein 1 light chain 3 (MAP1LC3, hereafter LC3) to the lipid phosphatidylethanolamine [21–23]. The double-membrane structure containing the cargo is closed, forming the completed autophagosome, which then fuses with endocytic or lysosomal vesicles, leading to the degradation of the components [24–26].

In the 1990s, a phosphatidylinositol 3-kinase (PI3K), Vps34p, was first identified as an essential protein for autophagy in yeast [27], as it generates phosphatidylinositol 3-phosphate (PI(3)P), a lipid critical for the nucleation of autophagic vesicles [28,29]. While this initial work was performed in yeast, which only contain one PI3K, later research revealed eight human PI3K isoforms that differ in structure and substrate sensitivity [30,31]. Class III PI3K (phosphatidylinositol 3-kinase catalytic subunit type 3; PI3K-C3; VPS34), also a critical autophagy regulator in mammalian cells [32], shares a similar role to Vps34p in yeast, operating in an autophagy-specific complex with mammalian homologs VPS15, BECLIN1, and ATG14L [29,33,34].

To explore the function of all eight PI3K isoforms on autophagy, we performed a focused siRNA experiment. Along with PI3K-C3, as expected, we identified the Class II PI3K, phosphatidylinositol-4-phosphate 3-kinase catalytic subunit type 2 alpha (PI3K-C2α) as being critical for autophagy. Current research on PI3K-C2α has focused the enzyme’s role in vesicle trafficking [35] with PI3K-C2α having important roles for both clathrin-dependent and clathrin-independent internalization of vesicles, suggesting a dynamic role for the enzyme in the regulation of endocytosis [36–42]. While it has been suggested that PI3K-C2α may play a role in autophagy [34,43], the fundamental connections with PI3K-C2α to autophagy remains unclear.

It was originally believed that the Golgi apparatus and endoplasmic reticulum (ER) were the sole membrane sources for autophagic vesicles [44,45]. Indeed, these organelles contribute to phagophore formation [46–48], and it was later shown that other membranes, such as the mitochondria and plasma membrane, can also integrate directly into pre-autophagosomal structures [49–51]. Follow-up work revealed that ATG16L1- and ATG9-positive membrane sources from clathrin-mediated endocytosis (CME) coalesce in the recycling endosome before integrating into pre-autophagosomal vesicles [50,52–54]. This trafficking of ATG9-positive vesicles through the recycling endosome is required for autophagosome formation [55], and the formation of these pre-autophagosomal vesicles appears to be dependent on the formation of ATG9-complexes with the clathrin-adaptor proteins AP1/2 [17,19].

In this study, we show that PI3K-C2α acts as a positive regulator of autophagy. Analysis of the subcellular localization of PI3K-C2α reveals that the protein localizes with markers of endocytosis and pre-autophagosomal vesicles, including ATG9 and AP2. CME stimulation in conjunction with PI3K-C2α knockdown results in an enrichment of perinuclear vesicles that are positive for both clathrin and RAB11; these same markers accumulate upon the knockdown of ATG9. Based on these results, we conclude that PI3K-C2α is critical for coordinating the use of endosomes as an additional membrane source during autophagy, likely through interactions with ATG9.

## Results

### PI3K-C2α knockdown decreases autophagosome formation

**Fig 1:**
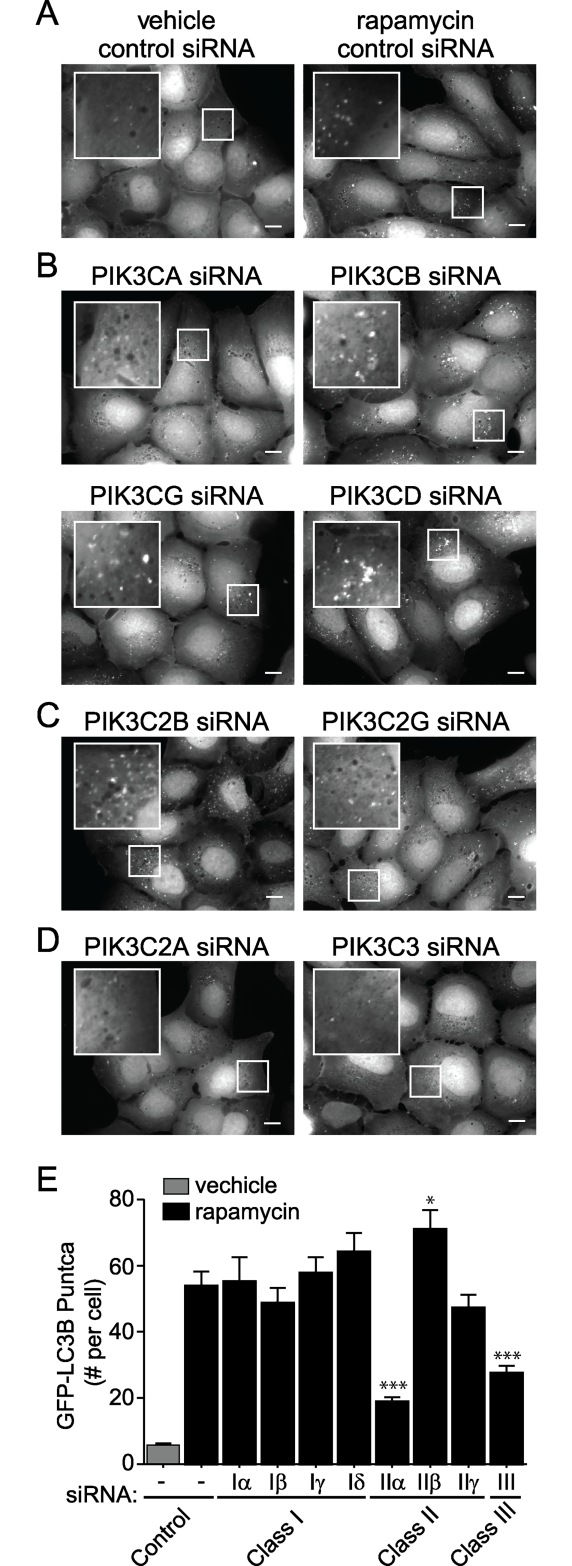
PI3K-C2α knockdown decreases autophagy. (A-E) U2OS cells stably expressing ptfLC3B were transfected with siRNAs directed against each of the eight PI3K isoforms for 48 hours. Cells were imaged with a 60× oil objective by fluorescent microscopy. Scale bars represent 10 μm. (A) Using a control siRNA, cells were treated with either vehicle or rapamycin for 6 hours to induce autophagy. (B-D) siRNA directed against each of the Class I PI3K isoforms *PIK3CA*, *PIK3CB*, *PIK3CG*, *PIK3CD* (B), *PIK3C2B* or *PIK3C2G* (C), and *PIK3C2A* or *PIK3C3* (D) and rapamycin treated for 6 hours. (E) Quantification of the number of autophagic puncta per cell in (A-D). Data represent means of n ≥ 25 cells with standard error of the mean. Unpaired *t* test, comparing experimental to rapamycin control. *denotes p < 0.05, ***denotes p < 0.001.

U2OS cells stably expressing LC3B fused to a tandem (EGFP and mRFP) fluorescent tag (ptfLC3-U2OS) [56–58] were transfected with control siRNA and treated with rapamycin, an mTORC1 inhibitor and autophagy inducer, for 6 hours. Rapamycin-treated cells showed increased EGFP-LC3B puncta when compared to vehicle control cells, consistent with increased autophagic vesicle formation and induction of autophagy (Fig 1A). To examine the isoform-specific role of the PI3Ks in autophagy, we knocked down each of the eight PI3K isoforms, treated cells with rapamycin as described above, and measured the formation of EGFP-LC3B autophagic puncta. Knockdown of each of the four Class I PI3Ks (*PIK3CA*, *PIK3CB*, *PIK3CG*, *PIK3CD*) and two of three Class II PI3Ks (*PIK3C2B* and *PIK3C2G*) (Fig 1B and 1C and S1 Fig) resulted in EGFP-LC3B puncta levels similar to control cells, suggesting that these PI3Ks do not dramatically regulate autophagy. Knockdown of *PIK3C3* (encoding the Class 3 PI3K, PI3K-C3, or VPS34), known to strongly impact autophagy signaling [29], or *PIK3C2A* (encoding the Class 2 PI3K, PI3K-C2α), decreased EGFP-LC3B puncta formation, suggesting that PI3K-C2α also plays an important role in autophagy (Fig 1D and S1 Fig). Specifically, control siRNA cells treated with rapamycin averaged 54 LC3-positive puncta per cell, while *PIK3C2A* and *PIK3C3* siRNA knockdown resulted in a 65% or 48% reduction, respectively (Fig 1E).

**Fig 2:**
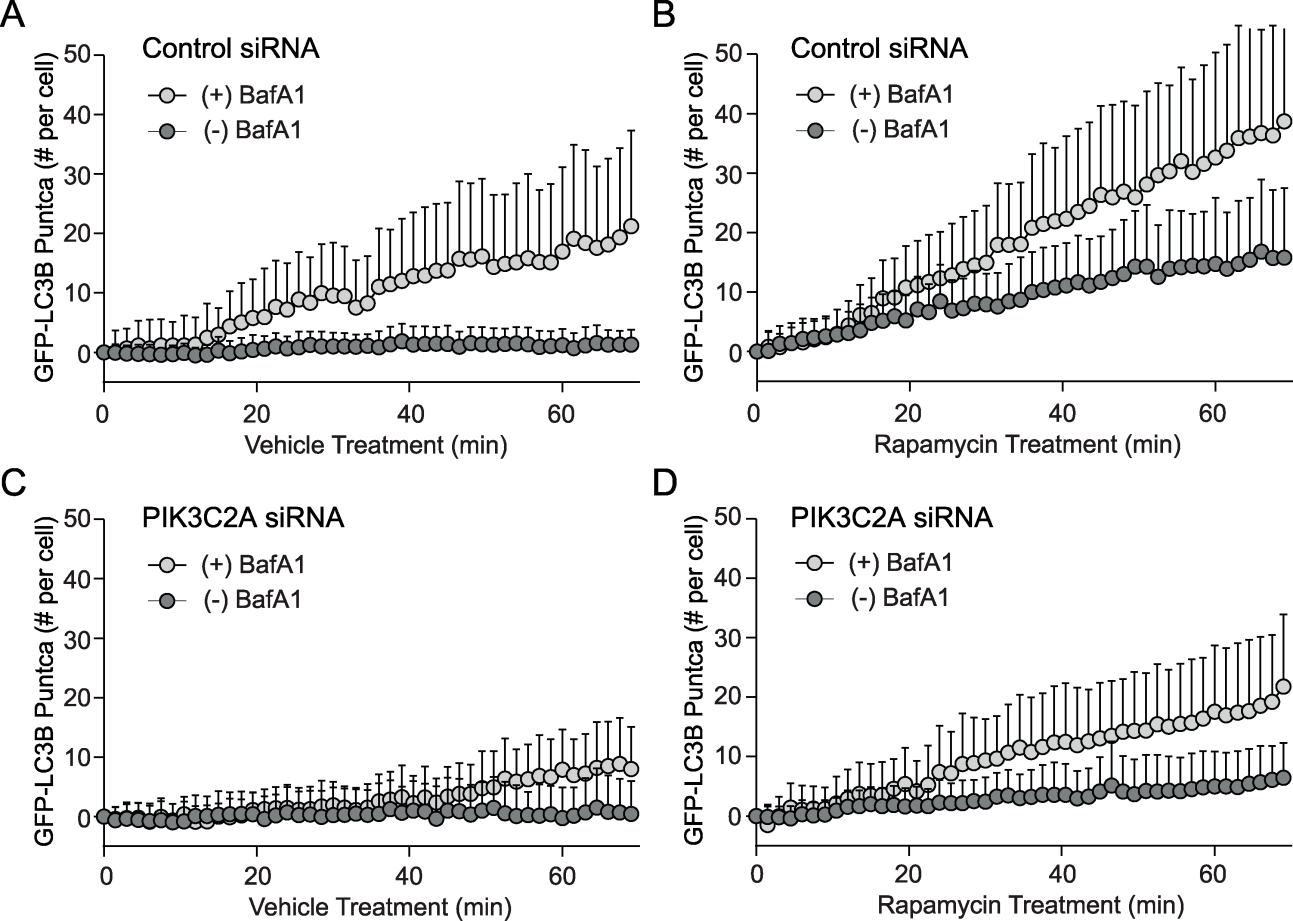
PI3K-C2α is a positive regulator of autophagy. (A-D) The number of GFP-LC3-II puncta that accumulated in the presence (light gray circles) or absence (dark gray circles) of BafA1 was plotted (t = 0 through t = 70 min). Values on the vertical axis represent mean numbers of puncta with adjustment such that the value at t = 0 is 0. Means were adjusted by subtracting the mean number of vesicles at t = 0. (A) Vehicle treated control siRNA cells, and (B) rapamycin induced autophagy in control siRNA conditions after rapamycin addition at time t = 0. (C, D) *PIK3C2A* siRNA knockdown (48 hours) and cells treated with vehicle control (C) or rapamycin (D). Bars represent standard deviations.

Previously, we developed a U2OS cellular system and image processing protocol to monitor both autophagosome synthesis and turnover in single cells using fluorescent images [59]. To characterize basal autophagy in U2OS cells, we imaged EGFP-LC3-positive puncta in single cells cultured in full-nutrient media with or without Bafilomycin A1 (BafA1), a V-ATPase inhibitor that prevents autophagosome turnover [60]. Following a short pretreatment period with either vehicle (–) or BafA1 (+), cells were imaged once every 1.5 min for 70 min. Representative images are shown in S2 Fig. As expected, vesicle counts increased for vehicle treated (Fig 2A) or rapamycin treated cells (Fig 2B), with the increase significantly higher in cells treated with rapamycin and BafA1. Next, we repeated these measurements of basal (Fig 2C) and induced autophagy (Fig 2D) after *PIK3C2A* knockdown. The decrease in both the absolute number of puncta and the rate of autophagosome formation per cell suggests that PI3K-C2α positively regulates autophagy.

### PI3K-C2α knockdown decreases autophagy and results in lipid droplet accumulation

**Fig 3:**
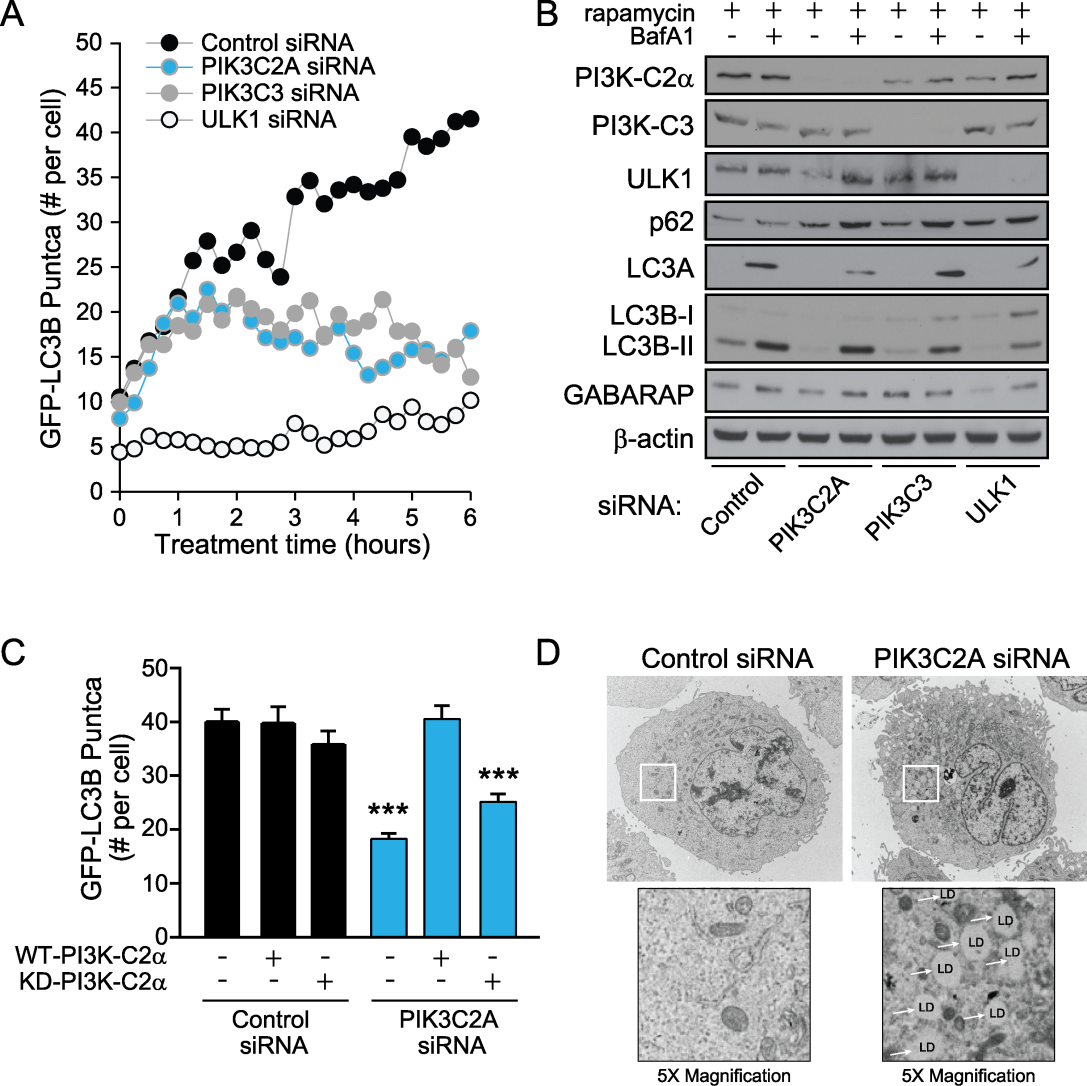
PI3K-C2α knockdown inhibits sustained autophagy and results in the formation of lipid droplets. (A) U2OS cells stably expressing ptfLC3B were transfected with siRNAs directed to *PIK3C2A*, *PIK3C3*, and *ULK1* for 48 hours. Live-cell imaging was carried out for six continuous hours after addition of rapamycin (50 nM) to measure changes in GFP-LC3 puncta accumulation. (B) U2OS cells were transfected with siRNAs directed to *PIK3C2A*, *PIK3C3*, and *ULK1* for 48 hours, and then treated with rapamycin (6 hours) to induce autophagy in the absence or presence of BafA1 (90 mins). ATG8 isoforms (LC3A, LC3B, and GABARAP) and p62 (SQSTM1) were examined to detect changes in autophagy. (C) U2OS cells stably expressing EGFP-LC3B were transfected with siRNAs directed to *PIK3C2A* for 48 hours. After 24 hours, siRNA-resistant wild-type protein (WT-PI3K-C2α) or siRNA-resistant kinase-dead protein (KD-PI3K-C2α) was transfected. After an additional 24 hours, cells were treated with rapamycin for 6 hours and number of puncta per cell counted from exogenous PI3K-C2α expressing cells. Representative images are shown in S3 Fig. Data represent means of n ≥ 25 cells with standard error of the mean. Unpaired *t* test, comparing experimental to control. ***denotes p < 0.001. (D) Loss of PI3K-C2α increases lipid droplets (LD) as measured by transmission electron microscopy. Insets are 5× magnifications.

To better understand the kinetics of autophagy and the role of PI3K-C2α and PI3K-C3 in autophagy, ptfLC3-U2OS cells were transfected with non-targeting (negative control), *ULK1* (positive control), *PIK3C2A*, or *PIK3C3* siRNA, and images acquired over a 6 hour time-period following the addition of rapamycin. *PIK3C2A* or *PIK3C3* knockdown resulted in a time-dependent decrease in the number of EGFP-LC3B positive puncta compared to the control cells (Fig 3A). At 1 hour, we observed that the level of autophagy with *PIK3C2A* and *PIK3C3* knockdown diverges from the control, and by 3 hours, the average puncta per cell for *PIK3C2A* and *PIK3C3* knockdown was reduced 48% and 39%, respectively. This divergence continued with sustained rapamycin treatment (6 hours), where we noticed a pronounced reduction in GFP-LC3B puncta per cell: 57% and 69% for *PIK3C2A* and *PIK3C3* knockdown, respectively. This indicated that both PI3K-C2α and PI3K-C3 are required for the sustained induction of autophagic vesicles. In comparison, knockdown of *ULK1*, a critical initiator of the autophagy process [15,61,62], resulted in an acute and sustained decrease in puncta formation per cell over the full time-course. Specifically, ULK1 knockdown yielded a 73% decrease in EGFP-LC3 puncta with acute rapamycin treatment (1 hour), and 76% decrease with sustained treatment (6 hours). This is consistent with previous reports that ULK1 is critical for acute induction of autophagic vesicles, as well as sustained induction [63].

To further explore the impact of PI3K-C2α loss on sustained autophagy, we examined endogenous protein levels by Western blotting for several autophagy markers following 6 hours rapamycin treatment (Fig 3B). Additionally, to measure autophagic turnover, cells were supplemented with or without BafA1 for the final 90 minutes of treatment. U2OS cells were transfected with siRNAs as described above. *PIK3C2A* knockdown decreased vesicle-lipidated LC3A (LC3A-II) under sustained rapamycin treatment with BafA1, consistent with a deficit in autophagy. We observed similar results with LC3B-II levels, although the differences were less pronounced. Interestingly, there was little change in the protein levels of GABARAP-II, an additional ATG8 isoform. In addition, we detected an accumulation of the autophagic cargo protein, p62/SQSTM1, following *PIK3C2A* knockdown which is also consistent with impaired autophagy. *PIK3C3* knockdown showed a modest decrease in LC3B-II levels and an accumulation of p62, demonstrating a partial defect in autophagy. For comparison, *ULK1* knockdown resulted in a distinct decrease in LC3A-II, LC3B-II, and GABARAP-II, as well as an accumulation of p62 under both treatment conditions, indicating a strong deficit in autophagy.

To validate that these knockdown studies, we performed siRNA rescue experiments to determine whether the low level of autophagy induction could be rescued with expression of exogenous wild-type (WT) or kinase-dead [64] PI3K-C2α. U2OS cells stably expressing EGFP-LC3B were transfected for 24 hours with either control siRNAs or siRNAs directed to *PIK3C2A*, followed by introduction of siRNA-resistant WT-PI3K-C2α or Kinase-Dead PI3K-C2α (KD-V5-PI3K-C2α) constructs [65] for an additional 24 hours. Indeed, cells expressing WT-PI3K-C2α in addition to *PIK3C2A* knockdown contained a similar number of puncta as control cells, compared to the knockdown alone (Fig 3C and S3 Fig). In contrast, cells expressing the KD-PI3K-C2α were unable to rescue the autophagy defect (Fig 3C), suggesting that the kinase activity of PI3K-C2α protein is required for its role in autophagy.

In addition to degrading proteins and bulk cytosol, autophagy supports lipid hydrolysis by releasing the content of lipid droplets to the lysosome for degradation. Moreover, autophagy inhibition is known to increase lipid storage in lipid droplets [66]. A striking result from *PIK3C2A* knockdown was the presence of abundant lipid droplets as observed by transmission electron microscopy (Fig 3D). Loss of PI3K-C2α resulted in both an increase in the number and size of lipid droplets. This is reminiscent of *ATG5* knockdown in cultured hepatocytes [66] and again highlights a role for PI3K-C2α in autophagy regulation.

### PI3K-C2α knockdown results in an accumulation of perinuclear endocytic vesicles

PI3K-C2α has an important role in endocytosis [36,38,41,65] regulated in part through interactions with its clathrin binding domain. Our next goal was to examine the impact of PI3K-C2α knockdown on CME under sustained autophagic conditions, as endocytosis can act to integrate membrane sources into the autophagy pathway [20,50,52,55,67,68]. U2OS cells were transfected with either control or *PIK3C2A* siRNAs for 48 hours and after 6 additional hours of rapamycin treatment, cells were treated with a transferrin Texas Red conjugate. Transferrin is an 80 kDa glycoprotein that binds to receptors on the cell surface which are then internalized through CME [69]. Once internalized, transferrin localizes to either peripheral or perinuclear endosomes. Peripheral transferrin can be associated with early endosomes that are EEA1 positive, while perinuclear transferrin is associated with recycling endosomes and is RAB11 positive [70]. In control cells, we observed an immediate coating of the cell with fluorescent transferrin-receptor complexes (S4A Fig). Transferrin conjugates were rapidly internalized in less than 5 minutes, and resulted in perinuclear vesicle fluorescence after 10 minutes. After 45 minutes, transferrin vesicles remained distributed throughout the cell, including the periphery, indicating continued trafficking of transferrin fluorescent dye-containing vesicles. In cells transfected with *PIK3C2A* siRNA, transferrin is coated on the cells and internalized in 5 minutes (S4B Fig), much like control siRNA cells. After 10 minutes, transferrin vesicles localized to the perinuclear region and throughout the cell, and by 45 minutes, the transferrin containing vesicles transitioned from a more homogenous cytosolic distribution to accumulation in the perinuclear region. This suggests that these vesicles are associated with the recycling endosome, but are not returning to the plasma membrane as observed under normal conditions (S4A Fig) [70]. To determine if this vesicle localization phenotype was unique to CME, cells were treated with CellMask Orange, a plasma membrane dye internalized by both clathrin-dependent and clathrin-independent pathways. Following 45 minutes of active endocytosis, we observed peripheral vesicle staining in control cells, while cells with *PIK3C2A* knockdown resulted in mostly perinuclear vesicle staining (S5 Fig); this suggests that the localization of vesicles is maintained in the presence of non-clathrin mediated vesicle internalization.

### PI3K-C2α colocalizes with endocytic vesicles

**Fig 4:**
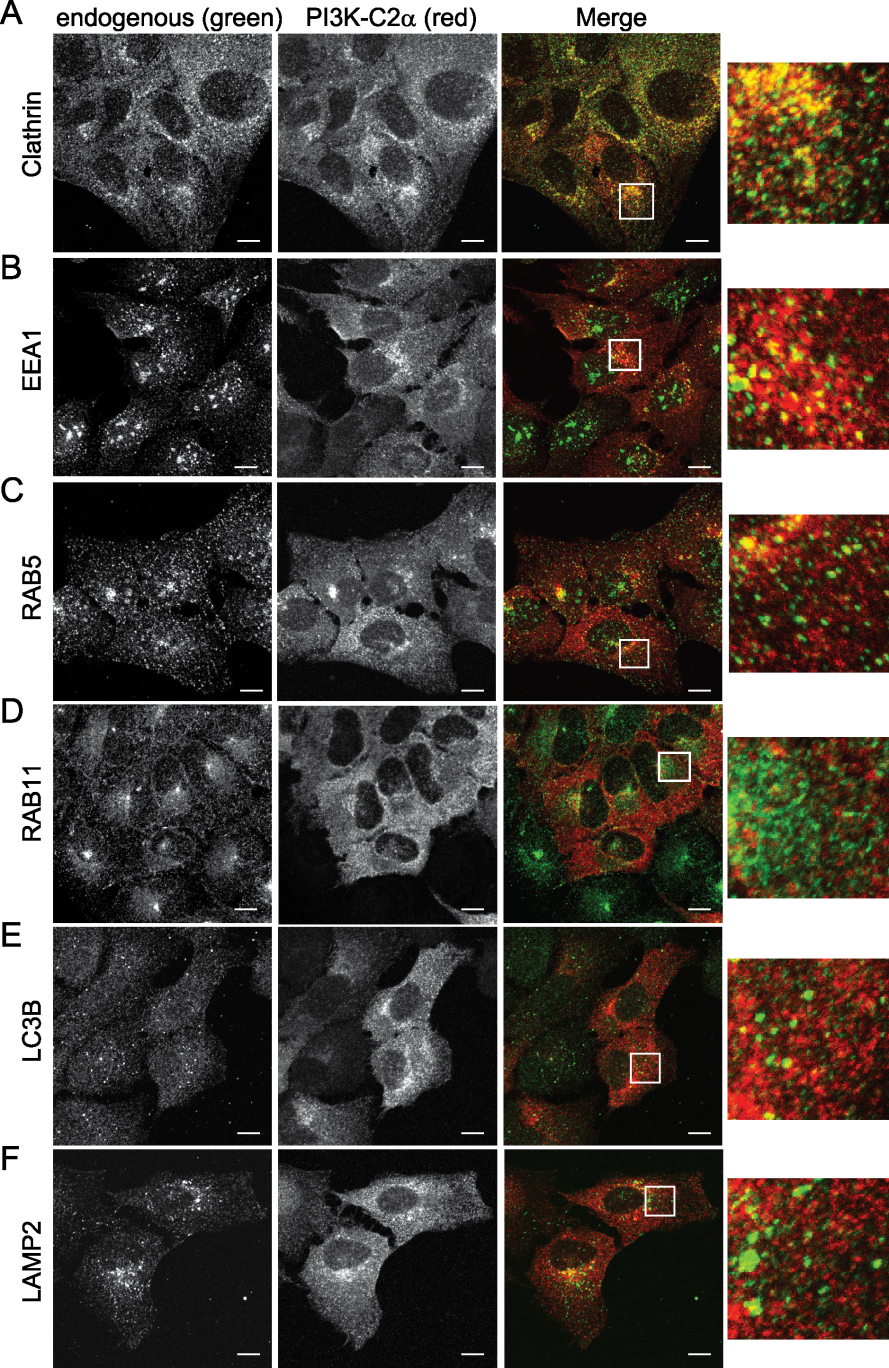
PI3K-C2α colocalizes with markers of early endocytosis. mCherry-PI3K-C2α expressing U2OS cell line was fixed and stained with primary antibodies against clathrin (A), EEA1 (B), RAB5 (C), RAB11 (D), LC3B (E), and LAMP2 (F). Secondary antibody (green) marks primary antibody staining. Cells were imaged with a 60× oil objective by confocal microscopy. Boxes are 5× magnifications of insets. Scale bar 10 μm.

To better understand the roles of PI3K-C2α in endocytosis and autophagy, we next explored whether PI3K-C2α colocalizes with endogenous markers. We found that mCherry-tagged PI3K-C2α is located on both perinuclear and peripheral vesicles (Fig 4). Next, we probed for endogenous markers in the endocytic pathway from early endocytosis clathrin coated vesicles (CCVs) to markers of vesicle maturation and turnover (Lysosomal Associated Membrane Protein 2, LAMP2). In cells containing both mCherry-PI3K-C2α (red vesicles) and endogenous marker (green vesicles) the amount of overlap (yellow) between vesicles can be calculated using the Pierson’s correlation coefficient (PCC) [71]. PI3K-C2α colocalized with endogenous clathrin (Fig 4A, PCC > 0.6), indicating PI3K-C2α at CCVs. Additionally, PI3K-C2α partially colocalized with endogenous EEA1 or RAB5 (Fig 4B and 4C, 0.6 > PCC > 0.3), suggesting a continued presence of PI3K-C2α at the early endosome. In contrast, PI3K-C2α showed a lower level of colocalization with endogenous RAB11, LC3B, or LAMP2 (Fig 4D–4F, PCC < 0.3), thus demonstrating a reduced presence at the recycling endosome, autophagosomes, or autolysosomes. Overall, these data suggest that PI3K-C2α is predominantly located at CCVs and early endosomes.

### PI3K-C2α fractionates and interacts with markers of endocytosis and autophagy

**Fig 5:**
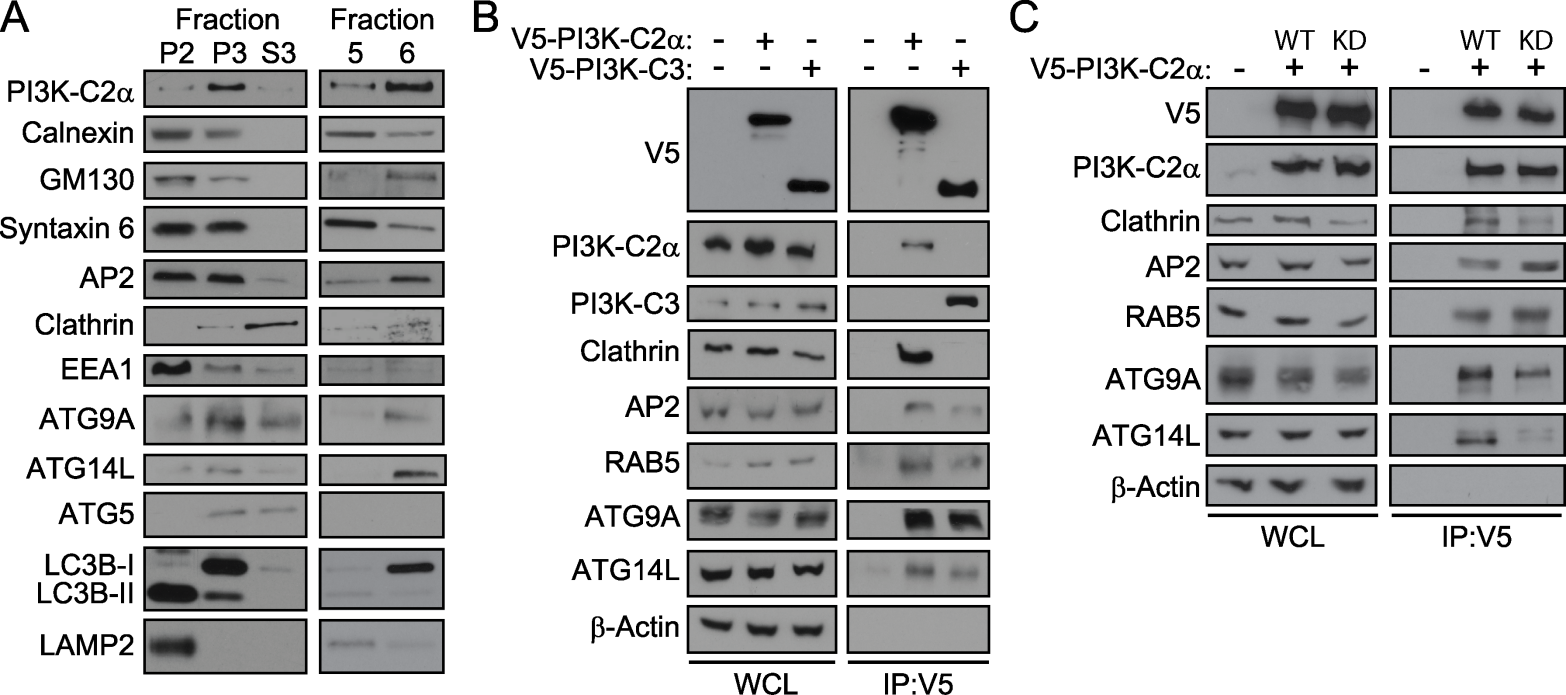
PI3K-C2α elutes and interacts with endocytic and autophagosomal markers. (A) U2OS fractions (P2, P3, and S3) from differential centrifugation (see S6 Fig) and OptiPrep gradient pellets (Fractions 5 and 6) were probed for markers of endocytosis and autophagy. (B) V5-PI3K-C2α or V5-PI3K-C3 were immunoprecipitated from 293FT cells and lysates probed for markers of endocytosis and autophagy. (C) Wild-Type (WT) or Kinase-Dead [64] V5-PI3K-C2α were immunoprecipitated from 293FT cells and lysates probed for markers of endocytosis and autophagy. (B, C) Whole cell lysates (WCL) were probed with the indicated antibodies as controls.

To further examine the subcellular localization of PI3K-C2α, we performed membrane fractionation experiments [48]. Briefly, lysed cells were centrifuged at increasing speeds, resulting in the collection of different cellular components in the pellet or supernatant (S6 Fig). Western blots were performed on samples P2, P3, and S3 (Fig 5A), and we observed that PI3K-C2α predominantly fractionated in the light membrane fraction P3 with the plasma membrane clathrin-adaptor protein AP2, the endosomal proteins clathrin and EEA1, and the early autophagosomal membrane precursors, ATG5, ATG9A, ATG14L, and LC3B-I. A previous report from Schekman and colleagues observed both cytosolic and membrane bound fractions of LC3B-I, and found that the levels of cytosolic versus membrane bound LC3-I can vary based on cell type or whether autophagy is defective [48]. To further resolve the protein components of the light membranes, we subjected P3 to an OptiPrep density gradient [48], removed sequential levels of the density gradient, and performed Western blot analysis (Fig 5A). We observed PI3K-C2α in fractions 5 and 6, along with plasma membrane marker (AP2), endosomal markers (clathrin and EEA1), and a portion of the *cis-*Golgi network marker, GM130 (most of which is distributed into P2 prior to the density gradient), as well as ATG9A, ATG14L, and LC3B-I.

Our next goal was to better understand differences and similarities in the PI3K-C2α and PI3K-C3 protein complexes. To do this, we performed co-immunoprecipitation of V5-PI3K-C2α and V5-PI3K-C3 expression constructs. Whole cell lysates were probed as a control for protein loading and to monitor the expression levels of endocytic markers (clathrin, AP1, AP2, RAB5, and RAB11) and autophagy markers (LC3A, LC3B, GABARAP, ATG7, ATG9A, ATG14, ATG16L1) (Fig 5B and S7 Fig). PI3K-C2α immunoprecipitated with clathrin, AP2, RAB5, ATG9A, and ATG14L; while PI3K-C3 also immunoprecipitated with these same proteins (Fig 5B). It is important to note that while PI3K-C2α and PI3K-C3 each immunoprecipitated with overlapping markers of endocytosis and autophagy, they did not immunoprecipitate with one another. Additionally, we immunoprecipitated either WT-PI3K-C2α or KD-PI3K-C2α to determine whether the kinase activity impacted complex formation or interactions, noting a decrease in association with clathrin, ATG9A, and ATG14L with PI3K-C2α lacking kinase activity (Fig 5C), suggesting catalytic activity may be important for the integrity of PI3K-C2α complexes.

### PI3K-C2α knockdown results in a RAB11 accumulation with transferrin vesicles

**Fig 6:**
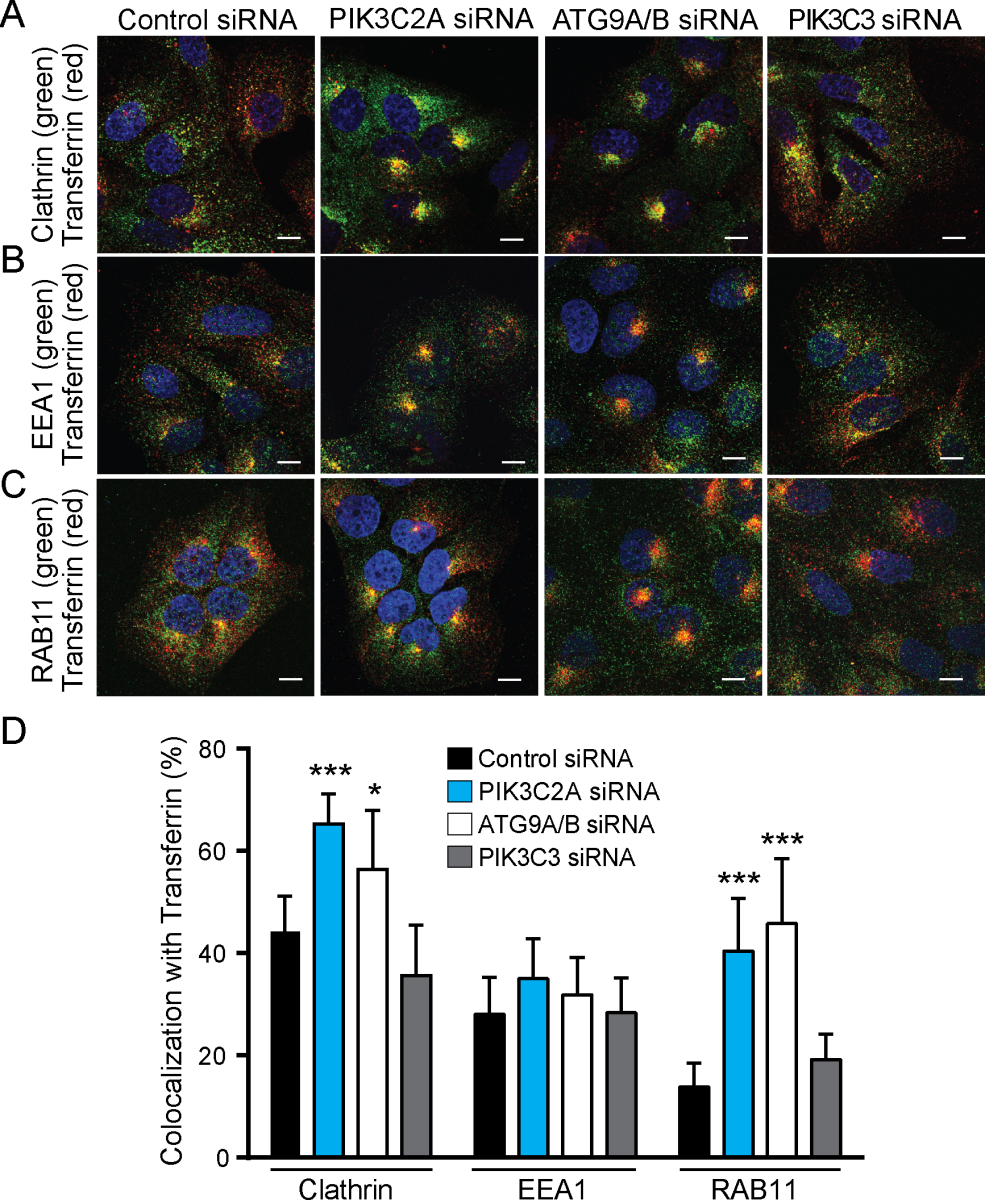
PI3K-C2α or ATG9 knockdown results in transferrin accumulation at the recycling endosome. U2OS cells were transfected with either control siRNAs or siRNAs directed to *PIK3C2A*, *ATG9A/B*, *or PIK3C3* for 48 hours. Following rapamycin treatment (6 hours), cells were incubated with Texas Red-conjugated transferrin, washed with fresh media, and returned to 37°C. Cells were stained with antibodies for endogenous clathrin (A), EEA1 (B), and RAB11 (C). Secondary antibody (green) marks primary antibody staining. Cells were imaged using confocal with a 60× oil objective. Scale bar shows 10 μm. (D) Percent colocalization of endogenous proteins with Texas Red-transferrin vesicles. Unpaired *t* test comparing experimental and control. *denotes p < 0.05, ***denotes p < 0.001.

**Fig 7:**
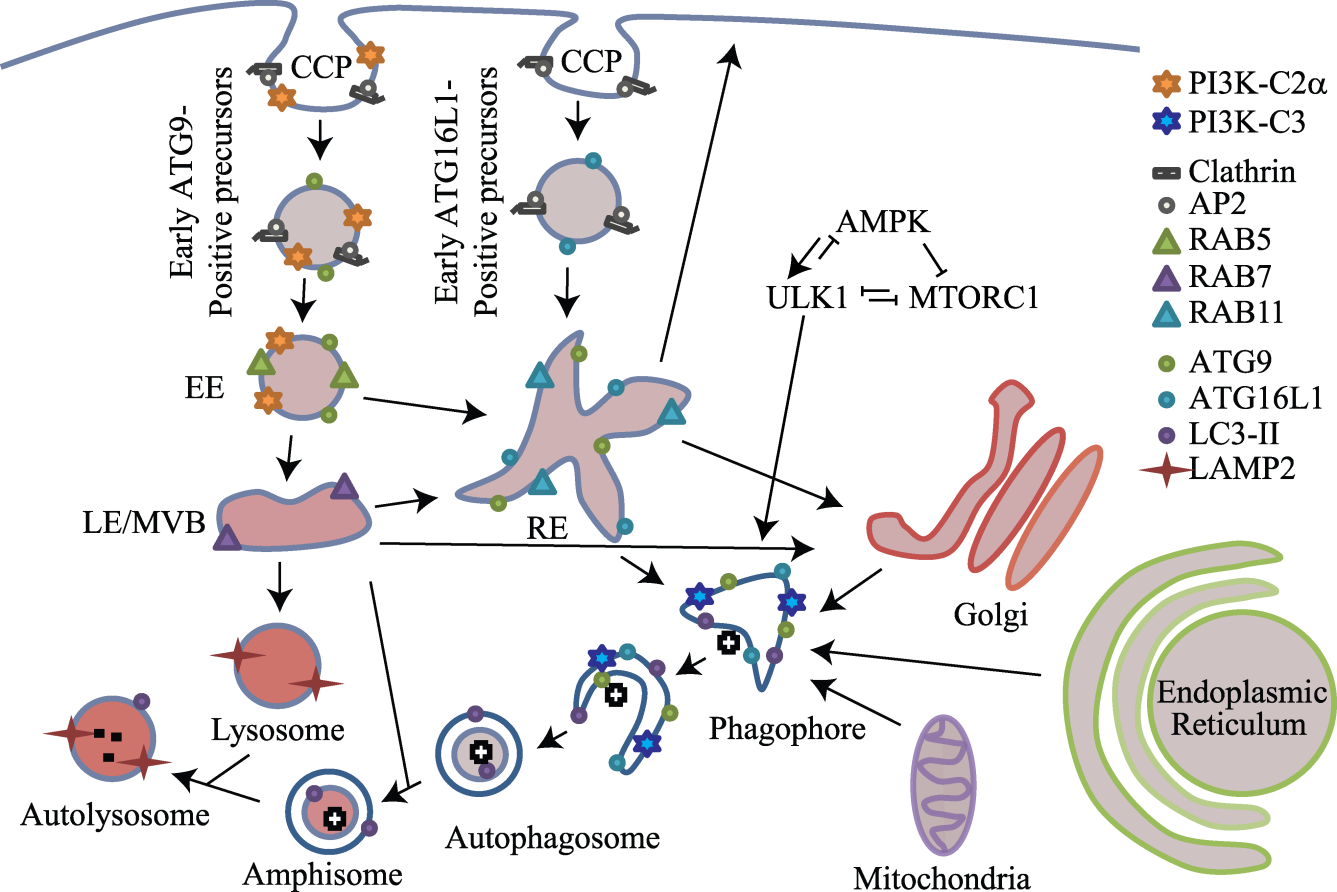
Proposed model for how PI3K-C2α may facilitate the integration of endocytic membrane sources into the autophagy pathway through the recycling endosome. Model highlighting PI3K-C2α localization in the endocytic and autophagy pathways. Abbreviations: CCP: clathrin-coated pit; EE: early endosome; LE: late endosome; MVB: multi-vesicular body; RE: recycling endosome.

To further investigate the effects of *PIK3C2A* knockdown on vesicular trafficking, we measured transferrin uptake and colocalization with markers of clathrin coated vesicles (Fig 6A), EEA1-positive early endosomes (Fig 6B), and RAB11-positive recycling endosomes (Fig 6C). Control siRNA cells displayed intermediate transferrin colocalization with clathrin (44%), EEA1 (28%), and less colocalization with RAB11 (14%). Consistent with earlier data (S3 Fig), *PIK3C2A* knockdown resulted in an accumulation of transferrin coated vesicles at the perinuclear recycling endosome with little peripheral spread in contrast with control cells. Furthermore, *PIK3C2A* knockdown cells displayed a significant increase in colocalization between transferrin and clathrin (p < 0.001), and between transferrin and RAB11 (p < 0.001). Next, due to the co-fractionation and co-immunoprecipitation of ATG9 with PI3K-C2α, we examined ATG9 loss by knocking down both *ATG9A* and *ATG9B* (S1C Fig) [51]. *ATG9A/B* knockdown cells displayed similar transferrin colocalization with clathrin, EEA1, and RAB11 as did *PIK3C2A* knockdown (Fig 6). Conversely, knockdown of *PI3KC3* displayed similar transferrin colocalization with clathrin, EEA1, and RAB 11 as did control. *PIK3C2A* or *ATG9A/B* knockdown resulted in: *1)* increased colocalization of transferrin with clathrin (65% colocalization with *PIK3C2A* knockdown, p < 0.05; 56% colocalization with *ATG9A/B* knockdown, p < 0.001) as compared to clathrin colocalization with control cells (44%); *2)* similar colocalization of transferrin with EEA1 (35% colocalization with *PIK3C2A* knockdown; 32% colocalization with *ATG9A/B* knockdown) as compared to control cells (28%); and *3)* increased colocalization of transferrin with RAB11 (p < 0.001), corresponding to 40% colocalization with *PIK3C2A* knockdown, 46% colocalization with *ATG9A/B* knockdown, as compared to 14% colocalization in control cells. To determine if the increased clathrin and RAB11 association resulted in colocalization of clathrin and RAB11 markers, we imaged transferrin, clathrin, and RAB11 within the same cell (S8 Fig). We found increased colocalization of clathrin and RAB11 within transferrin-positive vesicles (PCCs of 0.20, 0.43, 0.49 for control siRNA, *PIK3C2A* siRNA, *and ATG9A/B* siRNA, respectively). Taken together, this data suggests that PI3K-C2α and ATG9 may both be involved in the maturation of endocytic vesicles. Based on current literature surrounding ATG9 and endocytosis [50,52], we propose that *PIK3C2A* knockdown decreases autophagy by preventing the integration of endocytic membrane sources into the autophagy pathway through the recycling endosome (Fig 7).

## Discussion

Autophagy plays an important and context-dependent role in human diseases [4,8,72–74]. In order to better understand how to manipulate this complex process therapeutically, we must first determine how different proteins and pathways function in autophagy. To this end, we are interested in characterizing kinases and phosphatases that may serve as regulators of autophagy [57,58]. Here, we analyzed the eight PI3K isoforms in autophagy following knockdown and treatment with the allosteric MTORC1 inhibitor, rapamycin. In this setting, the autophagy assay measures MTORC1 dependent autophagy signaling and is suitable for the identification of both downstream effectors of MTORC1-induced autophagy (ULK1) and genes important for autophagy (*PIK3C2A*). Prior work has shown that Class I PI3Ks inhibit autophagy by activating AKT, which promotes MTOR signaling, thus suppressing autophagy [30,75,76]. In agreement with current literature, we find that PI3K-C2α and PI3K-C3 are positive regulators of autophagy while PIK3-C2β is a negative regulator of autophagy [29,34,43,77]. Here, we analyzed the functional consequences of an understudied PI3K family gene, *PIK3C2A*, to determine how the protein PI3K-C2α impacts the processes of endocytosis and autophagy. Using a combination of confocal microscopy, membrane fractionation, and immunoprecipitation, our results show that PI3K-C2α is present at both early endosomal and autophagosomal membranes, and for the first time, present a link for PI3K-C2α to autophagy and endocytosis.

While probing the kinetics of PI3K-C2α relative to the well characterized autophagy regulators, PI3K-C3 and ULK1 [12,31], we observed that these genes each have very different roles and significance in the autophagy pathway. Our results suggest that while ULK1 is critical for both acute and sustained autophagic induction, PI3K-C2α and PI3K-C3 are primarily required for more sustained autophagy. In addition, we highlight that knockdown of *PIK3C2A*, *PIK3C3*, or *ULK1* results in different levels of conjugation by the ATG8 mammalian homologs, LC3A, LC3B, and GABARAP. Consistent with previous studies [78,79], our results show that these genes have unique functions with regards to autophagic turnover.

While the literature supports a role for PI3K-C2α in the budding of CCVs, our results demonstrate an additional role for PI3K-C2α downstream of budding CCVs as far as the recycling endosome [41,65,80]. Elegant work by the Hirsch lab has shown that knockdown of PI3K-C2α leads to a more peripheral localization of RAB11 [81]; however, in their study, RAB11 is activated by a restricted pool of PtdIns3P localized to primary cilium. Therefore, these contrasting results emphasize how treatment conditions (rapamycin) and cell type can impact the roles of PI3K-C2α [82]. Here, the presence of PI3K-C2α, along with ATG9, is important for the maturation of CCVs. Because PI3K-C2α co-immunoprecipitated and fractionated with ATG9, and knockdown of either gene led to phenotypically similar endocytosis defects, our work further strengthens a connection between the endocytosis and autophagy pathways. Rubinsztein and colleagues reported that endocytic membrane sources can integrate directly into autophagosomal vesicles through ATG9 and ATG16L1 positive endosomes, which coalesce in the recycling endosome [50,52]; while Dikic and colleagues performed additional work on ATG9 vesicles, reporting that ATG9 interacts with the clathrin adaptor, AP2, and that AP2-positive CCVs are required for proper ATG9 localization, establishing a direct connection between ATG9 and CCVs [19]. This work also demonstrate an interaction between ATG9, AP2, and RAB5 [19], all of which were present in our co-immunoprecipitation experiments with PI3K-C2α, suggesting that PI3K-C2α may be an additional component of this complex. Importantly, this complex has been shown to regulate ATG9 localization and integration of these membrane sources into the autophagy pathway through the recycling endosome [19].

In many instances, internalized transferrin passes through early endosomes to the recycling endosome before transport back to the plasma membrane; although some reports show this is not always linear, as transferrin can bypass the recycling endosome [70]. We show that PI3K-C2α has a role in the recycling of internalized transferrin receptor through endosome maturation at the recycling endosome. *PIK3C2A* knockdown prevents the transferrin receptor from being transported back to the cell surface, likely due to defective maturation as the RAB11-postive endosomes remain clathrin-positive. Based on the current literature, we would predict that the condensed perinuclear RAB11 is maintained as an inactive state [81,83]. We further show that *PIK3C3* knockdown does not result in an accumulation of RAB11 vesicles, emphasizing that PI3K-C2α and PI3K-C3 have different roles in endocytosis. A role for PI3K-C2α in CCV and endosome maturation could clarify many reported signaling phenotypes, such as altered insulin signaling [84] and decreased TGFβ signaling [42] with *PIK3C2A* knockdown, if PI3K-C2α loss results in the sequestering of these different receptors due to incomplete endocytic maturation. Mutations in the clathrin binding and kinase domains of PI3K-C2α are important for clathrin localization [65], and a better understanding of these domains in autophagy and endocytosis remains to be elucidated. Important future studies, focused on Class II PI3K isoforms, will reveal cell type and context dependent cues for targeting the autophagic and endocytic pathways.

## Materials and methods

### Antibodies, reagents, and plasmids

The following antibodies from Cell Signaling Technology were used for Western blotting: ATG5 (12994S), ATG7 (8558S), ATG16L1 (8089S), β-Actin (3700S), Calnexin (2679S), Clathrin Heavy Chain (4796S), EEA1 (2411S), GABARAP (13733S), GM130 (12480S), LC3A (4599S), RAB5 (3547S), RAB11 (5589S), ULK1 (8054S), and PI3K-C3 (4263S). Western blot antibodies from other sources included ATG9A (Abcam, ab108338; GeneTex, GTX128427), ATG14 (MBL International, PD026), AP1µ2 (Santa Cruz, sc-69446), AP2M1 (Origene, TA503020), LAMP2 (Abcam, H4B4), LC3B (Sigma, L7543), p62/SQSTM1 (Abnova, H00008878-M0 1), PI3K-C2α (Santa Cruz, sc-365290), and V5 (mouse: Invitrogen, R96025; rabbit: Sigma, V8137), Secondary antibodies include HRP-linked mouse (GE Healthcare, NA931) or rabbit (GE Healthcare, NA934) IgG. Antibodies used for immunofluorescence microscopy as follows: clathrin heavy chain (Cell Signaling Technology, 4796S), clathrin light chain (Santa Cruz, sc-12735), EEA1 (Cell Signaling Technology, 2411S), LAMP2 (Abcam, H4B4), LC3B (MBL International, M152-3), RAB5 (Cell Signaling Technology, 3547S), RAB11 (Cell Signaling Technology, 5589S), and V5 (Invitrogen, R96025). Secondary antibodies include mouse AF350 (Thermo Fisher, A-11045), mouse AF488 (Thermo Fisher, A-11001), rabbit AF488 (Thermo Fisher, A-11008), and mouse AF546 (Thermo Fisher, A-11030).

The following reagents were purchased commercially: rapamycin (LC Labs, 553210), Bafilomycin A1 (AG Scientific, B-1183), DMSO (Sigma Aldrich, D2650), formaldehyde (Thermo Fisher, 28908), and Hoechst 33342 (Invitrogen, H1399).

Plasmids used include ptfLC3 (Addgene, 21074) (Kimura, 2007), EGFP-LC3B (Addgene, 11546). V5-PI3K-C2α and mCherry-PI3K-C2α were generated from *PIK3C2A* full length cDNA (Open Biosystems, NM_002645) by PCR amplification and TOPO cloning using pCR8/GW-TOPO entry vectors (Invitrogen, K2500-20) and pRK7-nV5/ccdB and pcDNA3.1 (Invitrogen, V790-20) destination vectors, respectively. V5-PI3K-C3 was generated from ORF (BC053651) by cloning into pRK7 by way of SalI/EcoRI using a PCR primer to introduce an N-terminal V5 tag. Kinase-Dead PI3K-C2α (KD-V5-PI3K-C2α) was generated using a QuikChange Lightning Multi Site-Directed Mutagenesis Kit (Agilent, 210515), following the manufacturer’s protocol and using primers to introduce point mutations,K1138A, D1157A, and D1250A, as previously characterized [65].

### Cell culture and transfection

Cells were grown in antibiotic-free media supplemented with 10% fetal bovine serum (CellGro, 35-101-CV) at 37°C and 5% CO_2_, using McCoy’s 5A medium (Invitrogen, 16600–082) for U2OS (ATCC, HTB-96) cells and DMEM medium (Invitrogen, 11995–065) for 293FT (ATCC, PTA-5077) cells. Cell cultures were maintained for at least one week before performing experiments, and were passaged a maximum number of 20 times.

siRNA transfections were performed at a concentration of 25 nM per siRNA using 2 μL/mL of Oligofectamine transfection reagent (Invitrogen, 12252011) for 48 hours. DNA transfections were performed for 24 hours using a concentration of 5 μg/mL DNA using 1:3 μg-DNA:μl-lipid FuGENE HD (Promega, E2311).

U2OS cells stably expressing mCherry-PI3K-C2α were generated using a lentiviral expression system. A plasmid solution was made by first combining 4 μg of each lentiviral packaging plasmid (pLP1, pLP2, and pVSV-G), 4 μg of the expression vector, and 60 μL of 2 M CaCl_2_ in 440 μL sterile H_2_O. 500 μL of 2x HBSP buffer (1.5 mM Na_2_HPO_4_, 10 mM KCl, 280 mM NaCl, 12 mM Dextrose, 50 mM HEPES) was transferred to a 15 mL conical and the plasmid solution was added, dropwise while mixing. The solution was added to a 10 cm plate of 293FT cells at 90% confluency, and after 48 hours the media was collected, spun at 1,000 g for 10 minutes to remove dead cells and the supernatant was filtered to harvest the lentivirus. A fresh culture of U2OS cells were infected by adding lentivirus to a 10 cm dish of cells with 8 μg/ml polybrene (Sigma, TR-1003-G). After 24 hours, the media was changed and polyclonal cells were selected in 500 μg/mL G418 (Gibco, 11811031) containing media.

### siRNA sequences

Control siRNA was AllStars Negative Control siRNA (Qiagen, SI03650318) and two or four gene-specific sequences (from Qiagen) used in experiments:

***PIK3CA***: CTGAGTCAGTATAAGTATATA, CTCCGTGAGGCTACATTAATA, CTCTGAGTCAGTATAAGTATA, AAGCTTTAGAATAATGCGCAA.
***PIK3CB***: CCCTTCGATAAGATTATTGAA, TCGGGAAGCTACCATTTCTTA, TGGGCGGTGGATTCACAGATA, TACGTTCGAGAATATGCTGTA.
***PIK3CG***: ATCGAAGTTTGCAGAGACAAA, CACCTTTACTCTATAACTCAA, AAGTATGACGTCAGTTCCCAA, CAAACTCACGTCTGCAACTAA.
***PIK3CD***: CCGGTCACGCATGAAGGCAAA, CGCCGTGATCGAGAAAGCCAA, TGCGTGCGCGTTATTTATTTA, CACGGGCACTGTGCGCAGTAA.
***PIK3C2A***: CAAGATGGTCGAATCAAGGAA, TTGAAGAGAGATCGACAGCAA, TACCCACTAATTGCATTGGAA, ACCGAGCAGTAGATCAAGTAA.
***PIK3C2B***: GAGGGAGGAGCTAAACGGTTA, CACTGTAGACTTGCTTATCTA, GTGGACTATGATGGTATCAAT, CTGCTAGAGCATCGGATCCTA.
***PIK3C2G***: CCAGATCAAGAAATTCGTAAA, CCCGTAGAAATGATAACTCCA, CAGCTACTGGGTGGGAGTATA, CTGTAGTGTCCCACTCGATAA.
***PIK3C3***: AACGCGAAAGTGGAAATCGTA, TCGGTTGGTGCATCTAATGAA, ATCAACGTCCAGCTTAAGATA, CATGGACAAGCTGTTACGGAA.
***ULK1***: CGCGCGGTACCTCCAGAGCAA, TGCCCTTTGCGTTATATTGTA.
***ATG9A***: CTGGATCCACCGGCTTATCAA, CACAAACGTGAGCTGACAGAA.
***ATG9B***: CAGCCGCGGCCTGGCGCTCAA, CAGGTTCTGCACGTCTTCTAT.

### Autophagy inhibition screen

U2OS cells expressing ptfLC3 were seeded at 2.0x10^4^ cells per well in 24 round-well plates on glass coverslips (Fisher, 12-545-81) and transfected with siRNAs to each of the eight PI3K isoforms, as described above. After 48 hours, cells were treated with 0.1% v/v DMSO control or 50 nM rapamycin in DMSO for 6 hours, and fixed with 4% formaldehyde in 1x PBS, permeabilized with 0.2% Triton X-100 in 1x PBS, stained with appropriate primary and secondary antibodies, and stained with Hoechst. Cells were imaged using an ECLIPSE Ti (Nikon) epifluorescent microscope at 60× magnification using an oil immersion lens. FITC (Excitation 465–495) and DAPI (Excitation 340–380) channels were captured for each image.

Images were analyzed using NIS-Elements Software (Nikon). Individual cells were manually outlined to specify regions of interest (ROIs) for analysis. Images were deconvolved using a *fast-deconvolution* and the background was removed using the *detect background* tool. Intensity thresholding of the FITC channel resulted in the quantification of puncta per cell which was analyzed using GraphPad Prism (n ≥ 25 cells per condition). Significance was determined using an unpaired *t* test using control and experimental siRNA knockdown.

### Immunoblotting

U2OS cells were lysed in 10 mM KPO_4_, 1 mM EDTA, 10 mM MgCl_2_, 5 mM EGTA, 50 mM bis-glycerophosphate, 0.5% NP40, 0.1% Brij35, 0.1% sodium deoxycholate, 1 mM NaVO_4_, 5 mM NaF, 2 mM DTT, and complete protease inhibitors (Sigma, P8340-5ML). Proteins were resolved by SDS-PAGE, transferred to PVDF (for low molecular weight proteins like LC3) or nitrocellulose membranes, blocked with 4% w/v non-fat dry milk in 1x TBS-T (TBS with 0.05% Tween 20), and probed with primary antibodies in blocking buffer at 4°C overnight followed by secondary antibodies in blocking buffer for 45 minutes at room temperature. Proteins were detected by chemiluminescence using ECL (100 nM Tris, pH 6.8, luminol (1.25 mM), p-coumaric acid (198 μM), H_2_O_2_ (0.009%)) or SuperSignal West Femto maximum sensitivity substrate (Thermo Fisher, 34095).

### Live-cell microscopy

U2OS cells expressing ptfLC3 were seeded at 3.15x10^4^ cells per chamber in 4 compartment glass-bottom dishes (Greiner Bio, 627870) and transfected with siRNA pools directed to non-targeting control, *PIK3C2A*, *PIK3C3*, or *ULK1*, as described above. 48 hours later, the dish was transferred to a Nikon ECLIPSE Ti fitted with a live cell chamber for maintaining cells at 37°C and 5% CO_2_ during imaging. Three locations in each well were selected for imaging with a 60× oil submersion lens. 0.1% v/v DMSO control or rapamycin were added (50 nM in DMSO) and cells were imaged in the FITC channel (Excitation 465–495) every 10 minutes for 6 hours. Images were analyzed as described above (n ≥ 10 cells per condition). Significance was determined using an unpaired *t* test using control and experimental siRNA knockdown.

### Rescue of siRNA phenotype

U2OS cells expressing EGFP-LC3B were seeded at 20,000 cells per well in 24 well round-bottom plates on glass coverslips and transfected with siRNA pools against non-targeting control or *PIK3C2A* as described above. After 24 hours, cells were transfected with V5-PI3K-C2α or KD-V5-PI3K-C2α as described above. Cells were treated with rapamycin (50 nM in DMSO) for 6 hours and then fixed and stained as described above. Coverslips were imaged on an A1plus-RSi scanning confocal microscope (Nikon) using 403, 488, and 561 excitation lasers and a 60× oil immersion lens. Images were analyzed as described above (n ≥ 25 cells per condition). Significance was determined using an unpaired *t* test using control and experimental siRNA knockdown.

### Transmission electron microscopy

U2OS cells were transfected with control or *PIK3C2A* siRNAs for 48 hours, cells were collected and resuspended in 2% glutaraldehyde fixative (Sigma-Aldrich, G5882). Cell pellets were embedded in 2% agarose, postfixed in osmium tetroxide, and dehydrated with an acetone series. Samples were infiltrated and embedded in Poly/Bed 812 resin, polymerized at 60°C for 24 hours, 70 nm sections generated with a Power Tome XL (Boeckeler Instruments), and placed on copper grids. Cells were examined for lipid droplets using a JEOL 100C× Transmission Electron Microscope at 100 kV with the electron microscopy services performed by the Michigan State University Center for Advanced Microscopy (East Lansing, MI).

### Transferrin localization

U2OS cells were seeded at 20,000 cells per well in 24 well round-bottom plates on glass coverslips and transfected with siRNA pools against non-targeting control, *PIK3C2A*, *ATG9A/B*, or *PIK3C3* as described above. After 48 hours, cells were treated with rapamycin (50 nM in DMSO) for 6 hours. Cells were then treated with 25 μg/mL transferrin from human serum, Texas Red conjugate (Thermo Fisher, T2875) at 4°C for 5 minutes, washed with fresh media, and returned to 37°C and 5% CO_2_ for 0–45 minutes. Cells were fixed and stained as described above. Coverslips were imaged on an A1plus-RSi scanning confocal microscope (Nikon) using 403, 488, and 561 excitation lasers and a 60× oil immersion lens. Images were analyzed using NIS-Elements Software. Individual cells were manually outlined and regions of interest were defined generating a binary ROI of transferrin localization. Overlap between endogenous antibodies and Texas Red-transferrin was calculated by quantifying the overlap of the green channel with the ROI. Results were analyzed using GraphPad Prism. Significance was determined using an unpaired *t* test using control and experimental siRNA knockdown.

### Colocalization microscopy

U2OS cells stably expressing mCherry-PI3K-C2α were seeded at 20,000 cells in 24 well round-bottom plates on glass coverslips. Cells were fixed and stained as described above. Coverslips were imaged on a Nikon A1plus-RSi scanning confocal microscope using 403, 488, and 561 nm excitation lasers and a 60× oil submersion lens. Images were analyzed using NIS-Elements Software. The average Pearson’s Correlation Coefficient (PCC) was calculated by drawing ROIs around n ≥ 25 cells and averaging the PCC per cell.

### Subcellular fractionation

U2OS cells were homogenized in lysosome lysis buffer (10 mM Tricine pH 7.2, 400 mM Sucrose, 1 mM EDTA, complete protease inhibitors, PhosSTOP (Roche, 04906845001)) using a Dounce homogenizer until > 90% of cells were broken. Lysates were spun at increasing speeds (1k x g, 3k x g, 25k x g, 100k x g) and pellets were collected at each step. The nuclear fraction and unbroken cells remained in the pellet (P0) after the initial spin at 1,000 x g and were discarded, while the supernatant underwent an additional spin at 3,000 x g where organelles, such as the mitochondria, were then collected from the pellet (P1). This process was repeated at 25,000 x g to pellet heavy membranes, such as the Golgi and autophagosomes (P2), and again at 100,000 x g to isolate light membranes, such as the plasma membrane and pre-autophagosomal vesicles (P3). Cytosolic proteins remained in the supernatant from this final spin (S3). Cell pellets were re-suspended in B88 buffer (20 mM HEPES-KOH pH 7.2, 250 mM Sorbitol, 150 mM potassium acetate, and 5 mM Magnesium acetate, filtered). Total protein was quantified using a Bradford protein assay and 10–20% of total protein was analyzed using Western blot; remaining protein was re-pelleted. The remaining pellet of interest was re-suspended in lysosome lysis buffer with 19% OptiPrep solution (Sigma, D1556) and 2.3 M sucrose. In a SW 40 Ti Rotor tube (Beckman), a gradient of OptiPrep from 35% to 0% [60] was layered around the 19% OptiPrep sample by diluting the OptiPrep in dilution buffer (60 mM Tricine pH 7.2, 250 mM sucrose, and 6 mM EDTA). The gradient was spun at 150k x g for 3 hours. Ten consecutive fractions were removed from the top of the gradient by pipet, diluted in lysosome lysis buffer, and pelleted at 100k x g. The resulting pellets were suspended in equal volume of B88 buffer and run analyzed using Western blot.

### Immunoprecipitation

293FT cells were seeded in 10 cm dishes and transfected with V5-PI3K-C2α, V5-PI3K-C3, or KD-V5-PI3K-C2α as described above. Dynabead-protein G (Thermo Fisher, 10003D) was washed in 1x PBS (using a magnetic field to contain the Dynabeads in order to remove buffer) and then suspended in buffer (1x PBS, 1% Triton X100) with 1 μg of V5 antibody at 4°C for 6 hours. Beads were washed 3x with 1x PBS and cells were lysed (see above) and pelleted; supernatant was added to the beads for an overnight incubation at 4°C (with a portion of the supernatant kept as a whole cell control). Beads were washed with buffer (1x PBS, 0.1% Triton X-100). Proteins were denatured from the beads by adding 2x sample buffer (4% SDS, 100 mM Tris-HCl pH 6.8, 0.02% (w/v) Bromophenol blue, 20% (v/v) Glycerol, 2% (v/v) βME) and boiling at 100°C for 10 minutes. Resulting supernatant was analyzed by Western blot.

## Supporting information

S1 Fig Western blot validation of protein knockdown. (A) U2OS cells were transfected with control siRNAs or siRNAs directed to each of the eight PI3K isoforms. (B) U2OS cells were transfected with control siRNAs or siRNAs directed to each of the four highest expressing PI3K isoforms in U2OS cells [85]. (C) U2OS cells were transfected with control siRNAs or siRNAs directed to *PIK3C2A*, *PIK3C3*, *ULK1*, or *ATG9A/B*.
(PDF)

S2 Fig PI3K-C2α knockdown suppresses basal and rapamycin-induced autophagic flux. U2OS cells were vehicle (A, B) or rapamycin treated (C, D) to quantify autophagic flux following control siRNA (A, C) or *PIK3C2A* (B, D) knockdown in the presence of BafA1. The number of GFP-LC3-II puncta that accumulated in the presence or absence of BafA1 is plotted in Fig 2.
(PDF)

S3 Fig WT-PI3K-C2α but not KD-PI3K-C2α rescues autophagy. U2OS cells stably expressing EGFP-LC3B were transfected with siRNAs directed to control (A) or *PIK3C2A* (B) for 48 hours. After 24 hours, siRNA-resistant wild-type protein (WT-PI3K-C2α) or siRNA-resistant kinase-dead protein (KD-PI3K-C2α) was transfected. After an additional 24 hours, cells were treated with rapamycin for 6 hours and puncta per cell counted from exogenous PI3K-C2α expressing cells (red). Data is quantified in Fig 3C.
(PDF)

S4 Fig PI3K-C2α knockdown results in a perinuclear accumulation of endosomes. U2OS cells were transfected with control siRNAs (A) or siRNAs directed to *PIK3C2A* (B) for 48 hours. Following rapamycin treatment (6 hours), cells were incubated with Texas Red-conjugated transferrin. Cells were washed with fresh media and returned to 37°C for the indicated amount of time (0, 5, 10, or 45 min.) before fixation. Fixed cells were imaged using confocal microscopy with a 60× oil objective. Scale bar 10 μm.
(PDF)

S5 Fig PI3K-C2α knockdown results in perinuclear accumulation of a plasma membrane stain. U2OS cells stably expressing EGFP-LC3B were transfected with siRNAs directed to control (A) or *PIK3C2A* (B). Following rapamycin treatment (6 hours), plasma membrane was uniformly labeled with CellMask Orange at 4°C and returned to 37°C for 45 minutes. Cells were imaged using confocal microscopy with a 60× oil objective. Boxes are 5× magnification of insets. Scale bars 10 μm.
(PDF)

S6 Fig Fractionation scheme detailing differential centrifugation steps. Cultured U2OS cells were homogenized and centrifuged in successive increasing speeds spins (100 ×g, 3000 ×g, 25000 ×g, and 100000 ×g). Supernatants (S1, S2, and S3) and pellets (P0, P1, P2, and P3) were collected at each step. Pellet P3 continued onto OptiPrep density gradient medium for PI3K-C2α detection (Fractions 5 and 6) and to test for markers co-eluting with PI3K-C2α. Bold text indicates pellets, supernatants, and fractions further examined in Fig 5.
(PDF)

S7 Fig Endocytosis and autophagy markers not detected in co-immunoprecipitation experiments. V5-PI3K-C2α or V5-PI3K-C3 were immunoprecipitated and resulting 293FT lysates probed for markers of endocytosis and autophagy. Whole cell lysates (WCL) were probed with the indicated antibodies. Data presented here corresponds to Fig 5B.
(PDF)

S8 Fig PI3K-C2α or ATG9 knockdown results in colocalization of clathrin and RAB11 in transferrin positive endosomes. U2OS cells were transfected with siRNAs directed to control (A), *PIK3C2A* (B), or *ATG9A/B* (C) for 48 hours. Following rapamycin treatment (6 hours), cells were treated with Texas Red-conjugated transferrin at 4°C. Cells were then washed with fresh media and returned to 37°C for 45 minutes. Cells were stained with antibodies for endogenous clathrin (blue) and RAB11 (green). Cells were imaged using confocal microscopy with a 60× oil objective. Scale bar 10 μm.
(PDF)

## References

[1] Kaur J, Debnath J (2015) Autophagy at the crossroads of catabolism and anabolism. Nature Reviews Molecular Cell Biology 16: 461–472. doi: 10.1038/nrm4024 26177004 10.1038/nrm4024

[2] Beaulaton J, Lockshin RA (1977) Ultrastructural study of the normal degeneration of the intersegmental muscles of Anthereae polyphemus and Manduca sexta (Insecta, Lepidoptera) with particular reference of cellular autophagy. Journal of Morphology 154: 39–57. doi: 10.1002/jmor.1051540104 915948 10.1002/jmor.1051540104

[3] Takeshige K, Baba M, Tsuboi S, Noda T, Ohsumi Y (1992) Autophagy in yeast demonstrated with proteinase-deficient mutants and conditions for its induction. The Journal of Cell Biology 119: 301–311. 1400575 10.1083/jcb.119.2.301 PMC2289660

[4] Levine B, Kroemer G (2008) Autophagy in the pathogenesis of disease. Cell 132: 27–42. doi: 10.1016/j.cell.2007.12.018 18191218 10.1016/j.cell.2007.12.018 PMC2696814

[5] Yang Z, Klionsky DJ (2010) Eaten alive: a history of macroautophagy. Nature Cell Biology 12: 814–822. doi: 10.1038/ncb0910-814 20811353 10.1038/ncb0910-814 PMC3616322

[6] Novikoff AB, Essner E (1962) Cytolysomes and mitochondrial degeneration. The Journal of Cell Biology 15: 140–146. 13939127 10.1083/jcb.15.1.140 PMC2106132

[7] Mortimore GE, Schworer CM (1977) Induction of autophagy by amino-acid deprivation in perfused rat liver. Nature 270: 174–176. 927529 10.1038/270174a0

[8] White E (2012) Deconvoluting the context-dependent role for autophagy in cancer. Nature Reviews Cancer 12: 401–410. doi: 10.1038/nrc3262 22534666 10.1038/nrc3262 PMC3664381

[9] Galluzzi L, Pietrocola F, Bravo-San Pedro JM, Amaravadi RK, Baehrecke EH, et al (2015) Autophagy in malignant transformation and cancer progression. The EMBO Journal 34: 856–880. doi: 10.15252/embj.201490784 25712477 10.15252/embj.201490784 PMC4388596

[10] Mizushima N (2010) The role of the Atg1/ULK1 complex in autophagy regulation. Current Opinion in Cell Biology 22: 132–139. doi: 10.1016/j.ceb.2009.12.004 20056399 10.1016/j.ceb.2009.12.004

[11] Gallagher LE, Williamson LE, Chan EY (2016) Advances in autophagy regulatory mechanisms. Cells 5. 10.3390/cells5020024 PMC4931673 27187479

[12] Wang B, Kundu M (2017) Canonical and noncanonical functions of ULK/Atg1. Current Opinion in Cell Biology 45: 47–54. doi: 10.1016/j.ceb.2017.02.011 28292700 10.1016/j.ceb.2017.02.011 PMC5678971

[13] Kim J, Kundu M, Viollet B, Guan KL (2011) AMPK and mTOR regulate autophagy through direct phosphorylation of Ulk1. Nature Cell Biology 13: 132–141. doi: 10.1038/ncb2152 21258367 10.1038/ncb2152 PMC3987946

[14] Alers S, Loffler AS, Wesselborg S, Stork B (2012) Role of AMPK-mTOR-Ulk1/2 in the regulation of autophagy: cross talk, shortcuts, and feedbacks. Molecular and Cellular Biology 32: 2–11. doi: 10.1128/MCB.06159-11 22025673 10.1128/MCB.06159-11 PMC3255710

[15] Young AR, Chan EY, Hu XW, Kochl R, Crawshaw SG, et al (2006) Starvation and ULK1-dependent cycling of mammalian Atg9 between the TGN and endosomes. Journal of Cell Science 119: 3888–3900. doi: 10.1242/jcs.03172 16940348 10.1242/jcs.03172

[16] Russell RC, Tian Y, Yuan H, Park HW, Chang YY, et al (2013) ULK1 induces autophagy by phosphorylating Beclin-1 and activating VPS34 lipid kinase. Nature Cell Biology 15: 741–750. doi: 10.1038/ncb2757 23685627 10.1038/ncb2757 PMC3885611

[17] Zhou C, Ma K, Gao R, Mu C, Chen L, et al (2017) Regulation of mATG9 trafficking by Src- and ULK1-mediated phosphorylation in basal and starvation-induced autophagy. Cell Research 27: 184–201. doi: 10.1038/cr.2016.146 27934868 10.1038/cr.2016.146 PMC5339848

[18] He C, Baba M, Klionsky DJ (2009) Double duty of Atg9 self-association in autophagosome biogenesis. Autophagy 5: 385–387. 19182520 10.4161/auto.5.3.7699 PMC2833293

[19] Popovic D, Dikic I (2014) TBC1D5 and the AP2 complex regulate ATG9 trafficking and initiation of autophagy. EMBO Reports 15: 392–401. doi: 10.1002/embr.201337995 24603492 10.1002/embr.201337995 PMC3989670

[20] Pavel M, Rubinsztein DC (2017) Mammalian autophagy and the plasma membrane. The FEBS Journal 284: 672–679. doi: 10.1111/febs.13931 27758042 10.1111/febs.13931

[21] Mizushima N, Sugita H, Yoshimori T, Ohsumi Y (1998) A new protein conjugation system in human. The counterpart of the yeast Apg12p conjugation system essential for autophagy. The Journal of Biological Chemistry 273: 33889–33892. 9852036 10.1074/jbc.273.51.33889

[22] Ichimura Y, Kirisako T, Takao T, Satomi Y, Shimonishi Y, et al (2000) A ubiquitin-like system mediates protein lipidation. Nature 408: 488–492. doi: 10.1038/35044114 11100732 10.1038/35044114

[23] Hanada T, Noda NN, Satomi Y, Ichimura Y, Fujioka Y, et al (2007) The Atg12-Atg5 conjugate has a novel E3-like activity for protein lipidation in autophagy. The Journal of Biological Chemistry 282: 37298–37302. doi: 10.1074/jbc.C700195200 17986448 10.1074/jbc.C700195200

[24] Ericsson JL (1969) Studies on induced cellular autophagy. II. Characterization of the membranes bordering autophagosomes in parenchymal liver cells. Experimental Cell Research 56: 393–405. 4309985 10.1016/0014-4827(69)90030-5

[25] Gordon PB, Seglen PO (1988) Prelysosomal convergence of autophagic and endocytic pathways. Biochemical and Biophysical Research Communications 151: 40–47. 3126737 10.1016/0006-291x(88)90556-6

[26] Klionsky DJ, Eskelinen EL, Deretic V (2014) Autophagosomes, phagosomes, autolysosomes, phagolysosomes, autophagolysosomes… wait, I'm confused. Autophagy 10: 549–551. doi: 10.4161/auto.28448 24657946 10.4161/auto.28448 PMC4091142

[27] Tsukada M, Ohsumi Y (1993) Isolation and characterization of autophagy-defective mutants of Saccharomyces cerevisiae. FEBS Letters 333: 169–174. 8224160 10.1016/0014-5793(93)80398-e

[28] Vanhaesebroek B, Leevers SJ, Panayotou G, Waterfield MD (1997) Phosphoinositide 3-kinases: A conserved family of signal transducers. Trends in Biochemical Sciences 22: 267–272. 9255069 10.1016/s0968-0004(97)01061-x

[29] Kihara A, Noda T, Ishihara N, Ohsumi Y (2001) Two distinct Vps34 phosphatidylinositol 3-kinase complexes function in autophagy and carboxypeptidase Y sorting in Saccharomyces cerevisiae. The Journal of Cell Biology 152: 519–530. 11157979 10.1083/jcb.152.3.519 PMC2196002

[30] Vanhaesebroeck B, Guillermet-Guibert J, Graupera M, Bilanges B (2010) The emerging mechanisms of isoform-specific PI3K signalling. Nature Reviews Molecular Cell Biology 11: 329–341. doi: 10.1038/nrm2882 20379207 10.1038/nrm2882

[31] Yu X, Long YC, Shen HM (2015) Differential regulatory functions of three classes of phosphatidylinositol and phosphoinositide 3-kinases in autophagy. Autophagy 11: 1711–1728. doi: 10.1080/15548627.2015.1043076 26018563 10.1080/15548627.2015.1043076 PMC4824607

[32] Kiel JA, Rechinger KB, van der Klei IJ, Salomons FA, Titorenko VI, et al (1999) The Hansenula polymorpha PDD1 gene product, essential for the selective degradation of peroxisomes, is a homologue of Saccharomyces cerevisiae Vps34p. Yeast 15: 741–754. doi: 10.1002/(SICI)1097-0061(19990630)15:9<741::AID-YEA416>3.0.CO;2-O 10398343 10.1002/(SICI)1097-0061(19990630)15:9<741::AID-YEA416>3.0.CO;2-O

[33] Reggiori F, Tucker KA, Stromhaug PE, Klionsky DJ (2004) The Atg1-Atg13 complex regulates Atg9 and Atg23 retrieval transport from the pre-autophagosomal structure. Developmental Cell 6: 79–90. 14723849 10.1016/s1534-5807(03)00402-7

[34] Devereaux K, Dall'Armi C, Alcazar-Roman A, Ogasawara Y, Zhou X, et al (2013) Regulation of mammalian autophagy by class II and III PI 3-kinases through PI3P synthesis. PLoS One 8. 10.1371/journal.pone.0076405 PMC3789715 24098492

[35] Campa CC, Franco I, Hirsch E (2015) PI3K-C2 alpha: One enzyme for two products coupling vesicle trafficking and signal transduction. FEBS Letters 589: 1552–1558. doi: 10.1016/j.febslet.2015.05.001 25979177 10.1016/j.febslet.2015.05.001

[36] Domin J, Pages F, Volinia S, Rittenhouse SE, Zvelebil MJ, et al (1997) Cloning of a human phosphoinositide 3-kinase with a C2 domain that displays reduced sensitivity to the inhibitor wortmannin. Biochem J 326 (Pt 1): 139–147. 9337861 10.1042/bj3260139 PMC1218647

[37] Prior IA, Clague MJ (1999) Localization of a class II phosphatidylinositol 3-kinase, PI3KC2alpha, to clathrin-coated vesicles. Molecular Cell Biology Research Communications 1: 162–166. doi: 10.1006/mcbr.1999.0126 10356367 10.1006/mcbr.1999.0126

[38] Domin J, Gaidarov I, Smith ME, Keen JH, Waterfield MD (2000) The class II phosphoinositide 3-kinase PI3K-C2alpha is concentrated in the trans-Golgi network and present in clathrin-coated vesicles. The Journal of Biological Chemistry 275: 11943–11950. 10766823 10.1074/jbc.275.16.11943

[39] Gaidarov I, Smith ME, Domin J, Keen JH (2001) The class II phosphoinositide 3-kinase C2alpha is activated by clathrin and regulates clathrin-mediated membrane trafficking. Molecular Cell 7: 443–449. 11239472 10.1016/s1097-2765(01)00191-5

[40] Krag C, Malmberg EK, Salcini AE (2010) PI3KC2 alpha, a class II PI3K, is required for dynamin-independent internalization pathways. Journal of Cell Science 123: 4240–4250. doi: 10.1242/jcs.071712 21081650 10.1242/jcs.071712

[41] Posor Y, Eichhorn-Gruenig M, Puchkov D, Schoneberg J, Ullrich A, et al (2013) Spatiotemporal control of endocytosis by phosphatidylinositol-3,4-bisphosphate. Nature 499: 233–+. doi: 10.1038/nature12360 23823722 10.1038/nature12360

[42] Aki S, Yoshioka K, Okamoto Y, Takuwa N, Takuwa Y (2015) Phosphatidylinositol 3-kinase class II alpha-isoform PI3K-C2alpha is required for transforming growth factor beta-induced Smad signaling in endothelial cells. The Journal of Biological Chemistry 290: 6086–6105. doi: 10.1074/jbc.M114.601484 25614622 10.1074/jbc.M114.601484 PMC4358250

[43] Behrends C, Sowa ME, Gygi SP, Harper JW (2010) Network organization of the human autophagy system. Nature 466: 68–76. doi: 10.1038/nature09204 20562859 10.1038/nature09204 PMC2901998

[44] de Duve C, Wattiaux R (1966) Function of lysosomes. Annual Review of Physiology 28: 435–492. doi: 10.1146/annurev.ph.28.030166.002251 5322983 10.1146/annurev.ph.28.030166.002251

[45] Juhasz G, Neufeld TP (2006) Autophagy: a forty-year search for a missing membrane source. PLoS Biology 4: e36 doi: 10.1371/journal.pbio.0040036 16464128 10.1371/journal.pbio.0040036 PMC1363699

[46] Hayashi-Nishino M, Fujita N, Noda T, Yamaguchi A, Yoshimori T, et al (2009) A subdomain of the endoplasmic reticulum forms a cradle for autophagosome formation. Nature Cell Biology 11: 1433–1437. doi: 10.1038/ncb1991 19898463 10.1038/ncb1991

[47] Yla-Anttila P, Vihinen H, Jokitalo E, Eskelinen EL (2009) 3D tomography reveals connections between the phagophore and endoplasmic reticulum. Autophagy 5: 1180–1185. 19855179 10.4161/auto.5.8.10274

[48] Ge L, Melville D, Zhang M, Schekman R (2013) The ER-Golgi intermediate compartment is a key membrane source for the LC3 lipidation step of autophagosome biogenesis. Elife 2. 10.7554/eLife.00947 PMC3736544 23930225

[49] Hailey DW, Rambold AS, Satpute-Krishnan P, Mitra K, Sougrat R, et al (2010) Mitochondria supply membranes for autophagosome biogenesis during starvation. Cell 141: 656–667. doi: 10.1016/j.cell.2010.04.009 20478256 10.1016/j.cell.2010.04.009 PMC3059894

[50] Ravikumar B, Moreau K, Jahreiss L, Puri C, Rubinsztein DC (2010) Plasma membrane contributes to the formation of pre-autophagosomal structures. Nature Cell Biology 12: 747–757. doi: 10.1038/ncb2078 20639872 10.1038/ncb2078 PMC2923063

[51] Lamb CA, Yoshimori T, Tooze SA (2013) The autophagosome: origins unknown, biogenesis complex. Nature Reviews Molecular Cell Biology 14: 759–774. doi: 10.1038/nrm3696 24201109 10.1038/nrm3696

[52] Puri C, Renna M, Bento CF, Moreau K, Rubinsztein DC (2013) Diverse autophagosome membrane sources coalesce in recycling endosomes. Cell 154: 1285–1299. doi: 10.1016/j.cell.2013.08.044 24034251 10.1016/j.cell.2013.08.044 PMC3791395

[53] Moreau K, Puri C, Rubinsztein DC (2015) Methods to analyze SNARE-dependent vesicular fusion events that regulate autophagosome biogenesis. Methods 75: 19–24. doi: 10.1016/j.ymeth.2014.11.005 25461811 10.1016/j.ymeth.2014.11.005 PMC4358838

[54] Takahashi Y, Tsotakos N, Liu Y, Young MM, Serfass J, et al (2016) The Bif-1-Dynamin 2 membrane fission machinery regulates Atg9-containing vesicle generation at the Rab11-positive reservoirs. Oncotarget 7: 20855–20868. doi: 10.18632/oncotarget.8028 26980706 10.18632/oncotarget.8028 PMC4991497

[55] Imai K, Hao F, Fujita N, Tsuji Y, Oe Y, et al (2016) Atg9A trafficking through the recycling endosomes is required for autophagosome formation. Journal of Cell Science 129: 3781–3791. doi: 10.1242/jcs.196196 27587839 10.1242/jcs.196196

[56] Kimura S, Noda T, Yoshimori T (2007) Dissection of the autophagosome maturation process by a novel reporter protein, tandem fluorescent-tagged LC3. Autophagy 3: 452–460. 17534139 10.4161/auto.4451

[57] Martin KR, Xu Y, Looyenga BD, Davis RJ, Wu CL, et al (2011) Identification of PTP sigma as an autophagic phosphatase. Journal of Cell Science 124: 812–819. doi: 10.1242/jcs.080341 21303930 10.1242/jcs.080341 PMC3039021

[58] Goodall ML, Wang T, Martin KR, Kortus MG, Kauffman AL, et al (2014) Development of potent autophagy inhibitors that sensitize oncogenic BRAF V600E mutant melanoma tumor cells to vemurafenib. Autophagy 10: 1120–1136. doi: 10.4161/auto.28594 24879157 10.4161/auto.28594 PMC4091172

[59] Martin KR, Barua D, Kauffman AL, Westrate LM, Posner RG, et al (2013) Computational model for autophagic vesicle dynamics in single cells. Autophagy 9: 74–92. doi: 10.4161/auto.22532 23196898 10.4161/auto.22532 PMC3542220

[60] Klionsky DJ, Abdelmohsen K, Abe A, Abedin MJ, Abeliovich H, et al (2016) Guidelines for the use and interpretation of assays for monitoring autophagy (3rd edition). Autophagy 12: 1–222. doi: 10.1080/15548627.2015.1100356 26799652 10.1080/15548627.2015.1100356 PMC4835977

[61] Roach PJ (2011) AMPK -> ULK1 -> autophagy. Molecular and Cellular Biology 31: 3082–3084. doi: 10.1128/MCB.05565-11 21628530 10.1128/MCB.05565-11 PMC3147606

[62] Chan EY, Kir S, Tooze SA (2007) siRNA screening of the kinome identifies ULK1 as a multidomain modulator of autophagy. The Journal of Biological Chemistry 282: 25464–25474. doi: 10.1074/jbc.M703663200 17595159 10.1074/jbc.M703663200

[63] Wong PM, Puente C, Ganley IG, Jiang X (2013) The ULK1 complex: sensing nutrient signals for autophagy activation. Autophagy 9: 124–137. doi: 10.4161/auto.23323 23295650 10.4161/auto.23323 PMC3552878

[64] Klionsky DJ, Abdalla FC, Abeliovich H, Abraham RT, Acevedo-Arozena A, et al (2012) Guidelines for the use and interpretation of assays for monitoring autophagy. Autophagy 8: 445–544. doi: 10.4161/auto.19496 22966490 10.4161/auto.19496 PMC3404883

[65] Gaidarov I, Zhao Y, Keen JH (2005) Individual phosphoinositide 3-kinase C2alpha domain activities independently regulate clathrin function. The Journal of Biological Chemistry 280: 40766–40772. doi: 10.1074/jbc.M507731200 16215232 10.1074/jbc.M507731200

[66] Singh R, Kaushik S, Wang Y, Xiang Y, Novak I, et al (2009) Autophagy regulates lipid metabolism. Nature 458: 1131–1135. doi: 10.1038/nature07976 19339967 10.1038/nature07976 PMC2676208

[67] Longatti A, Lamb CA, Razi M, Yoshimura S, Barr FA, et al (2012) TBC1D14 regulates autophagosome formation via Rab11- and ULK1-positive recycling endosomes. The Journal of Cell Biology 197: 659–675. doi: 10.1083/jcb.201111079 22613832 10.1083/jcb.201111079 PMC3365497

[68] Knaevelsrud H, Soreng K, Raiborg C, Haberg K, Rasmuson F, et al (2013) Membrane remodeling by the PX-BAR protein SNX18 promotes autophagosome formation. J Cell Biol 202: 331–349. doi: 10.1083/jcb.201205129 23878278 10.1083/jcb.201205129 PMC3718966

[69] McMahon HT, Boucrot E (2011) Molecular mechanism and physiological functions of clathrin-mediated endocytosis. Nature Reviews Molecular Cell Biology 12: 517–533. doi: 10.1038/nrm3151 21779028 10.1038/nrm3151

[70] Sheff D, Pelletier L, O'Connell CB, Warren G, Mellman I (2002) Transferrin receptor recycling in the absence of perinuclear recycling endosomes. The Journal of Cell Biology 156: 797–804. doi: 10.1083/jcb.20111048 11877458 10.1083/jcb.20111048 PMC2173326

[71] Dunn KW, Kamocka MM, McDonald JH (2011) A practical guide to evaluating colocalization in biological microscopy. American Journal of Physiology—Cell Physiology 300: C723–742. doi: 10.1152/ajpcell.00462.2010 21209361 10.1152/ajpcell.00462.2010 PMC3074624

[72] Yue ZY, Jin SK, Yang CW, Levine AJ, Heintz N (2003) Beclin 1, an autophagy gene essential for early embryonic development, is a haploinsufficient tumor suppressor. Proceedings of the National Academy of Sciences of the United States of America 100: 15077–15082. doi: 10.1073/pnas.2436255100 14657337 10.1073/pnas.2436255100 PMC299911

[73] Qu XP, Yu J, Bhagat G, Furuya N, Hibshoosh H, et al (2003) Promotion of tumorigenesis by heterozygous disruption of the beclin 1 autophagy gene. Journal of Clinical Investigation 112: 1809–1820. doi: 10.1172/JCI20039 14638851 10.1172/JCI20039 PMC297002

[74] Lum JJ, Bauer DE, Kong M, Harris MH, Li C, et al (2005) Growth factor regulation of autophagy and cell survival in the absence of apoptosis. Cell 120: 237–248. doi: 10.1016/j.cell.2004.11.046 15680329 10.1016/j.cell.2004.11.046

[75] Wu YT, Tan HL, Shui G, Bauvy C, Huang Q, et al (2010) Dual role of 3-methyladenine in modulation of autophagy via different temporal patterns of inhibition on class I and III phosphoinositide 3-kinase. The Journal of Biological Chemistry 285: 10850–10861. doi: 10.1074/jbc.M109.080796 20123989 10.1074/jbc.M109.080796 PMC2856291

[76] Zhai C, Cheng J, Mujahid H, Wang H, Kong J, et al (2014) Selective inhibition of PI3K/Akt/mTOR signaling pathway regulates autophagy of macrophage and vulnerability of atherosclerotic plaque. PLoS One 9: e90563 doi: 10.1371/journal.pone.0090563 24599185 10.1371/journal.pone.0090563 PMC3944201

[77] Marat AL, Wallroth A, Lo WT, Muller R, Norata GD, et al (2017) mTORC1 activity repression by late endosomal phosphatidylinositol 3,4-bisphosphate. Science 356: 968–972. doi: 10.1126/science.aaf8310 28572395 10.1126/science.aaf8310

[78] Shpilka T, Weidberg H, Pietrokovski S, Elazar Z (2011) Atg8: an autophagy-related ubiquitin-like protein family. Genome Biology 12: 226 doi: 10.1186/gb-2011-12-7-226 21867568 10.1186/gb-2011-12-7-226 PMC3218822

[79] Lee YK, Lee JA (2016) Role of the mammalian ATG8/LC3 family in autophagy: differential and compensatory roles in the spatiotemporal regulation of autophagy. BMB Reports 49: 424–430. doi: 10.5483/BMBRep.2016.49.8.081 27418283 10.5483/BMBRep.2016.49.8.081 PMC5070729

[80] Zhao Y, Gaidarov I, Keen JH (2007) Phosphoinositide 3-kinase C2alpha links clathrin to microtubule-dependent movement. The Journal of Biological Chemistry 282: 1249–1256. doi: 10.1074/jbc.M606998200 17110375 10.1074/jbc.M606998200

[81] Franco I, Gulluni F, Campa CC, Costa C, Margaria JP, et al (2014) PI3K class II alpha controls spatially restricted endosomal PtdIns3P and Rab11 activation to promote primary cilium function. Developmental Cell 28: 647–658. doi: 10.1016/j.devcel.2014.01.022 24697898 10.1016/j.devcel.2014.01.022 PMC4042153

[82] Joo K, Kim CG, Lee MS, Moon HY, Lee SH, et al (2013) CCDC41 is required for ciliary vesicle docking to the mother centriole. Proceedings of the National Academy of Sciences of the United States of America 110: 5987–5992. doi: 10.1073/pnas.1220927110 23530209 10.1073/pnas.1220927110 PMC3625310

[83] Pasqualato S, Senic-Matuglia F, Renault L, Goud B, Salamero J, et al (2004) The structural GDP/GTP cycle of Rab11 reveals a novel interface involved in the dynamics of recycling endosomes. J Biol Chem 279: 11480–11488. doi: 10.1074/jbc.M310558200 14699104 10.1074/jbc.M310558200

[84] Leibiger B, Moede T, Paschen M, Yunn NO, Lim JH, et al (2015) PI3K-C2alpha knockdown results in rerouting of insulin signaling and pancreatic beta cell proliferation. Cell Reports 13: 15–22. doi: 10.1016/j.celrep.2015.08.058 26387957 10.1016/j.celrep.2015.08.058

[85] Elis W, Triantafellow E, Wolters NM, Sian KR, Caponigro G, et al (2008) Down-regulation of class II phosphoinositide 3-kinase alpha expression below a critical threshold induces apoptotic cell death. Molecular Cancer Research 6: 614–623. doi: 10.1158/1541-7786.MCR-07-0262 18403640 10.1158/1541-7786.MCR-07-0262

