# Supplementary figures and images for: PI3K-C2α knockdown decreases autophagy and maturation of endocytic vesicles
Source: PLoS One. 2017 Sep 14;12(9):e0184909. doi: 10.1371/journal.pone.0184909 (PMC5599018; doi:10.1371/journal.pone.0184909)

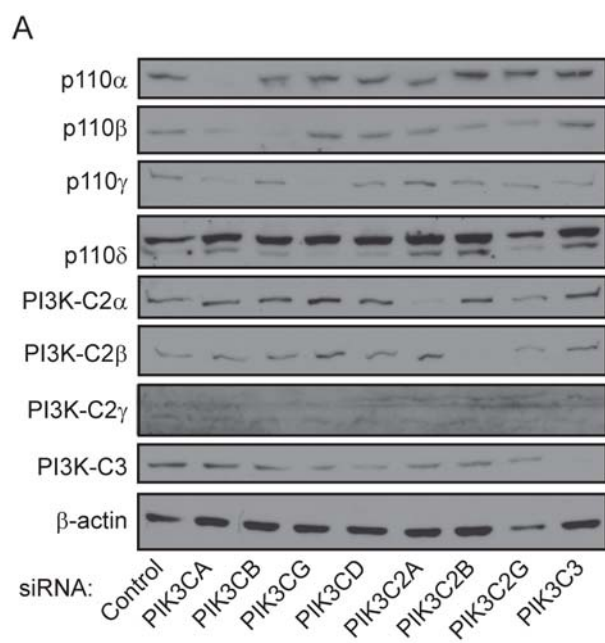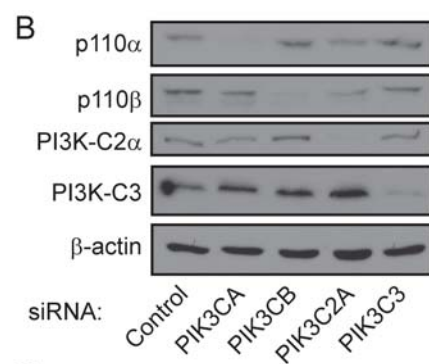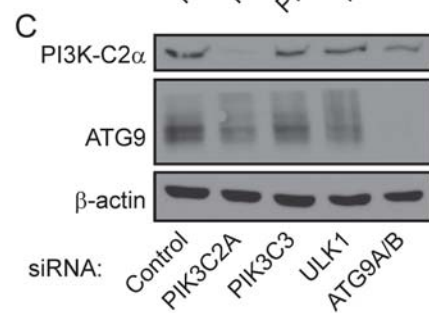

Supplement: S1 Fig — (A) U2OS cells were transfected with control siRNAs or siRNAs directed to each of the eight PI3K isoforms. (B) U2OS cells were transfected with control siRNAs or siRNAs directed to each of the four highest expressing PI3K isoforms in U2OS cells [85]. (C) U2OS cells were transfected with control siRNAs or siRNAs directed to PIK3C2A, PIK3C3, ULK1, or ATG9A/B. (PDF) [file pone.0184909.s001.pdf]

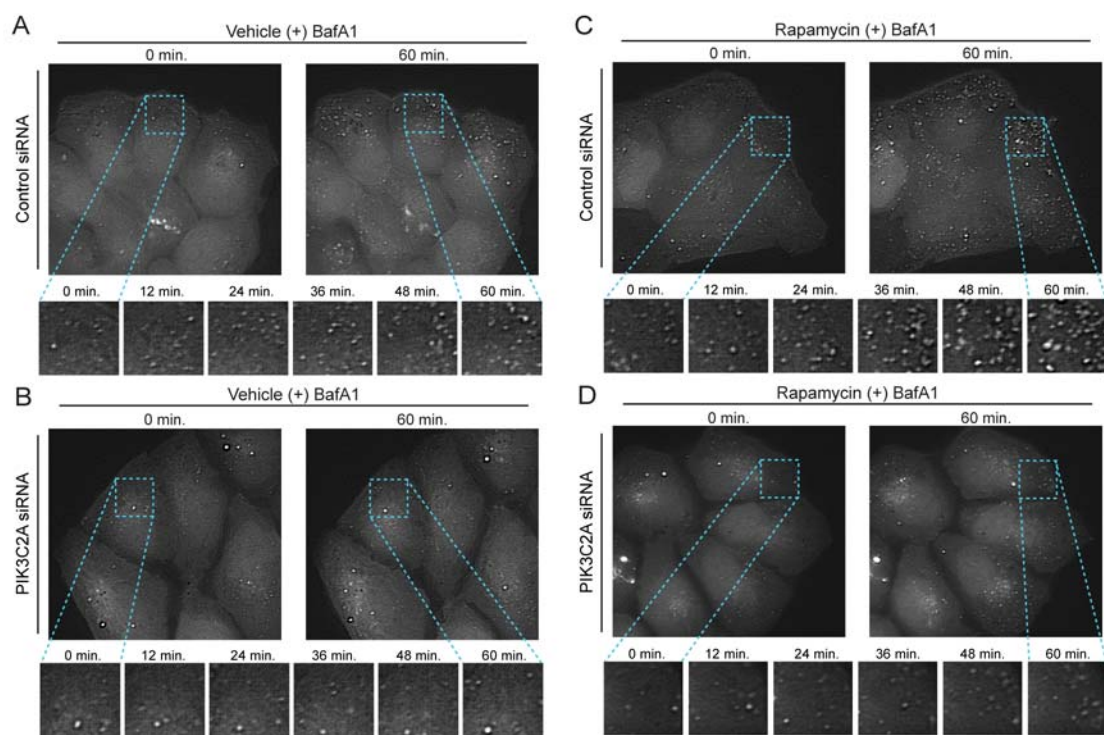

Supplement: S2 Fig — U2OS cells were vehicle (A, B) or rapamycin treated (C, D) to quantify autophagic flux following control siRNA (A, C) or PIK3C2A (B, D) knockdown in the presence of BafA1. The number of GFP-LC3-II puncta that accumulated in the presence or absence of BafA1 is plotted in Fig 2. (PDF) [file pone.0184909.s002.pdf]

A

Control siRNA

WT-PI3K-C2 $\alpha$ 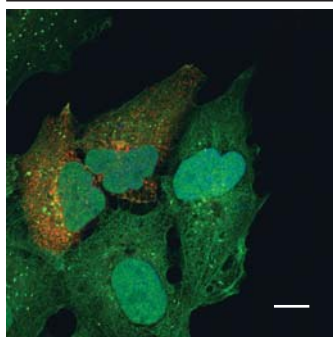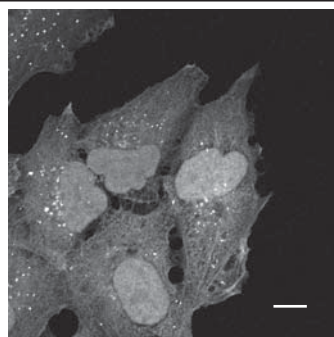KD-PI3K-C2 $\alpha$ 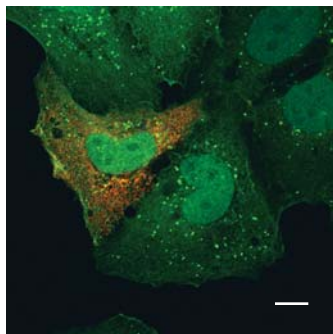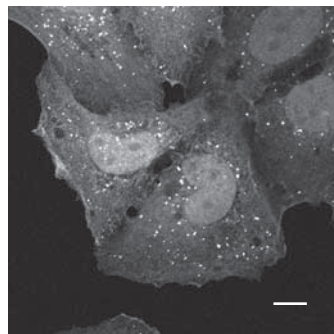

B

PIK3C2A siRNA

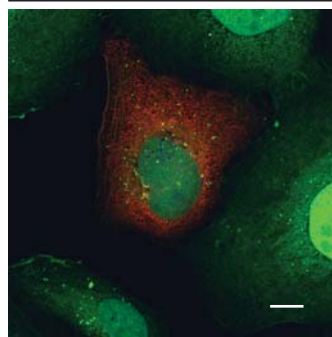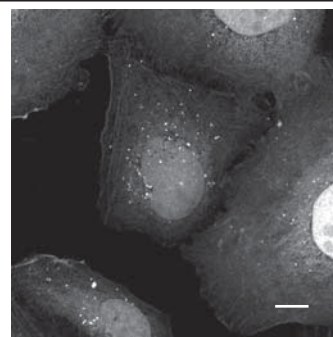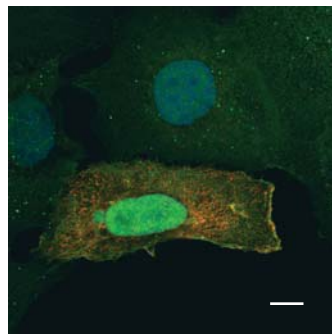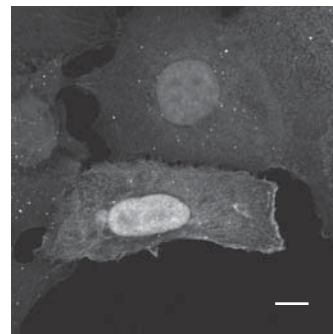

Supplement: S3 Fig — U2OS cells stably expressing EGFP-LC3B were transfected with siRNAs directed to control (A) or PIK3C2A (B) for 48 hours. After 24 hours, siRNA-resistant wild-type protein (WT-PI3K-C2α) or siRNA-resistant kinase-dead protein (KD-PI3K-C2α) was transfected. After an additional 24 hours, cells were treated with rapamycin for 6 hours and puncta per cell counted from exogenous PI3K-C2α expressing cells (red). Data is quantified in Fig 3C. (PDF) [file pone.0184909.s003.pdf]

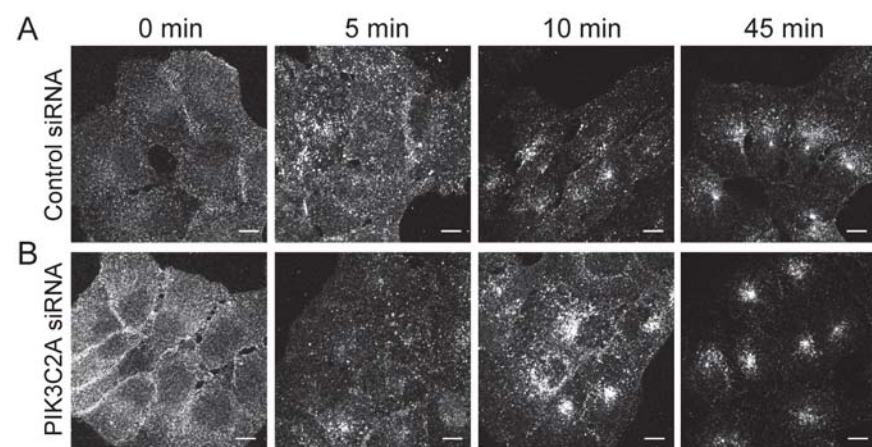

Supplement: S4 Fig — U2OS cells were transfected with control siRNAs (A) or siRNAs directed to PIK3C2A (B) for 48 hours. Following rapamycin treatment (6 hours), cells were incubated with Texas Red-conjugated transferrin. Cells were washed with fresh media and returned to 37°C for the indicated amount of time (0, 5, 10, or 45 min.) before fixation. Fixed cells were imaged using confocal microscopy with a 60× oil objective. Scale bar 10 μm. (PDF) [file pone.0184909.s004.pdf]

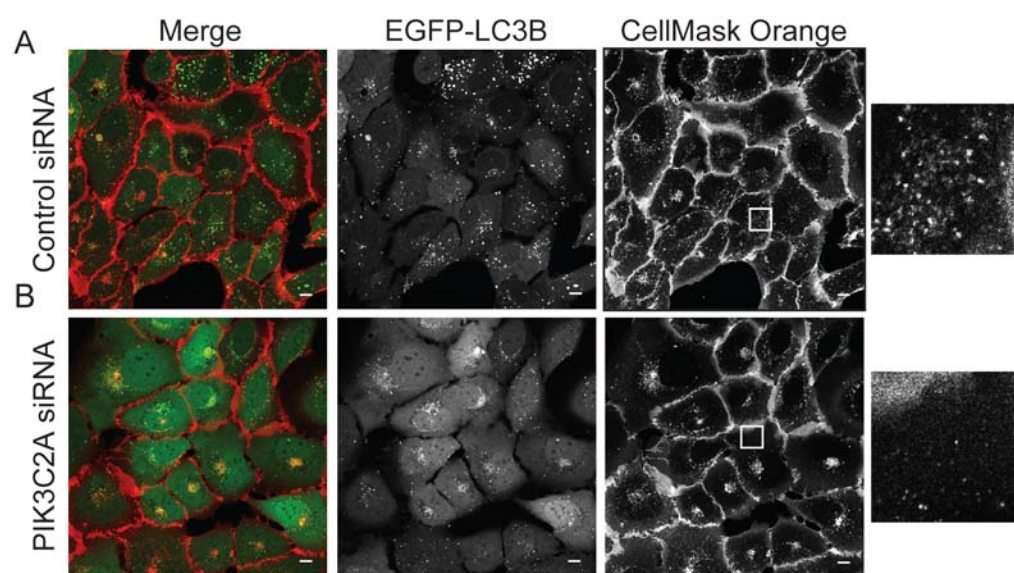

Supplement: S5 Fig — U2OS cells stably expressing EGFP-LC3B were transfected with siRNAs directed to control (A) or PIK3C2A (B). Following rapamycin treatment (6 hours), plasma membrane was uniformly labeled with CellMask Orange at 4°C and returned to 37°C for 45 minutes. Cells were imaged using confocal microscopy with a 60× oil objective. Boxes are 5× magnification of insets. Scale bars 10 μm. (PDF) [file pone.0184909.s005.pdf]

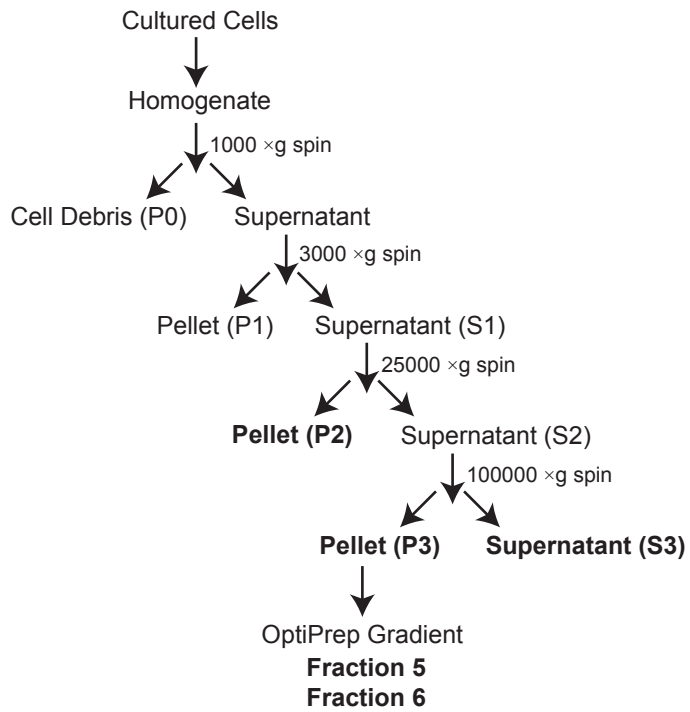

Supplement: S6 Fig — Cultured U2OS cells were homogenized and centrifuged in successive increasing speeds spins (100 ×g, 3000 ×g, 25000 ×g, and 100000 ×g). Supernatants (S1, S2, and S3) and pellets (P0, P1, P2, and P3) were collected at each step. Pellet P3 continued onto OptiPrep density gradient medium for PI3K-C2α detection (Fractions 5 and 6) and to test for markers co-eluting with PI3K-C2α. Bold text indicates pellets, supernatants, and fractions further examined in Fig 5. (PDF) [file pone.0184909.s006.pdf]

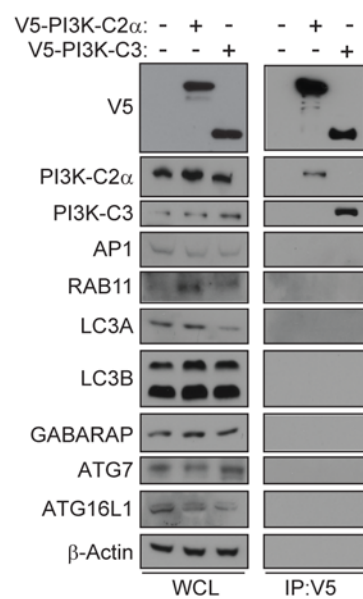

Supplement: S7 Fig — V5-PI3K-C2α or V5-PI3K-C3 were immunoprecipitated and resulting 293FT lysates probed for markers of endocytosis and autophagy. Whole cell lysates (WCL) were probed with the indicated antibodies. Data presented here corresponds to Fig 5B. (PDF) [file pone.0184909.s007.pdf]

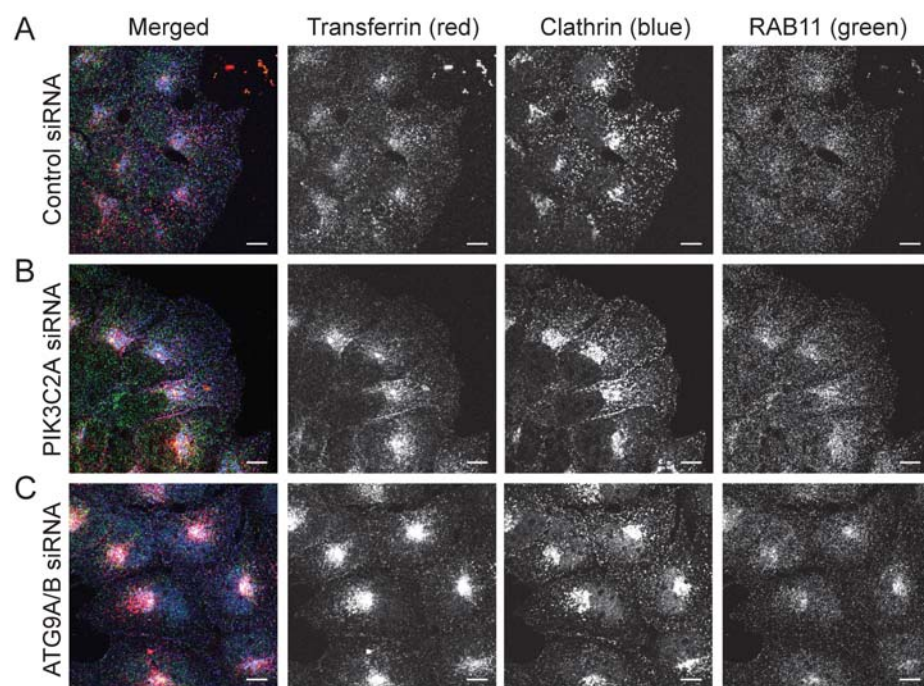

Supplement: S8 Fig — U2OS cells were transfected with siRNAs directed to control (A), PIK3C2A (B), or ATG9A/B (C) for 48 hours. Following rapamycin treatment (6 hours), cells were treated with Texas Red-conjugated transferrin at 4°C. Cells were then washed with fresh media and returned to 37°C for 45 minutes. Cells were stained with antibodies for endogenous clathrin (blue) and RAB11 (green). Cells were imaged using confocal microscopy with a 60× oil objective. Scale bar 10 μm. (PDF) [file pone.0184909.s008.pdf]
